# Dexamethasone modulates immature neutrophils and interferon programming in severe COVID-19

**DOI:** 10.1038/s41591-021-01576-3

**Published:** 2021-11-15

**Authors:** Sarthak Sinha, Nicole L. Rosin, Rohit Arora, Elodie Labit, Arzina Jaffer, Leslie Cao, Raquel Farias, Angela P. Nguyen, Luiz G. N. de Almeida, Antoine Dufour, Amy Bromley, Braedon McDonald, Mark R. Gillrie, Marvin J. Fritzler, Bryan G. Yipp, Jeff Biernaskie

**Affiliations:** 1 Department of Comparative Biology and Experimental Medicine, Faculty of Veterinary Medicine, University of Calgary, Calgary, AB Canada; 2 Calvin, Phoebe and Joan Snyder Institute for Chronic Diseases, Cumming School of Medicine, University of Calgary, Calgary, AB Canada; 3 Department of Critical Care Medicine, Cumming School of Medicine, University of Calgary, Calgary, AB Canada; 4 Department of Physiology and Pharmacology, University of Calgary, Calgary, AB Canada; 5 McCaig Institute for Bone and Joint Health, University of Calgary, Calgary, AB Canada; 6 Department of Pathology and Laboratory Medicine, University of Calgary, Calgary, AB Canada; 7 Department of Microbiology, Immunology and Infectious Diseases, University of Calgary, Calgary, AB Canada; 8 Department of Medicine, Cumming School of Medicine, University of Calgary, Calgary, AB Canada; 9 Department of Surgery, Cumming School of Medicine, University of Calgary, Calgary, AB Canada; 10 Hotchkiss Brain Institute, University of Calgary, Calgary, AB Canada; 11 Alberta Children’s Hospital Research Institute, University of Calgary, Calgary, AB Canada

**Keywords:** Target identification, Viral infection, Neutrophils, Immunotherapy, Innate immunity

## Abstract

Although critical for host defense, innate immune cells are also pathologic drivers of acute respiratory distress syndrome (ARDS). Innate immune dynamics during Coronavirus Disease 2019 (COVID-19) ARDS, compared to ARDS from other respiratory pathogens, is unclear. Moreover, mechanisms underlying the beneficial effects of dexamethasone during severe COVID-19 remain elusive. Using single-cell RNA sequencing and plasma proteomics, we discovered that, compared to bacterial ARDS, COVID-19 was associated with expansion of distinct neutrophil states characterized by interferon (IFN) and prostaglandin signaling. Dexamethasone during severe COVID-19 affected circulating neutrophils, altered IFN^active^ neutrophils, downregulated interferon-stimulated genes and activated IL-1R2^+^ neutrophils. Dexamethasone also expanded immunosuppressive immature neutrophils and remodeled cellular interactions by changing neutrophils from information receivers into information providers. Male patients had higher proportions of IFN^active^ neutrophils and preferential steroid-induced immature neutrophil expansion, potentially affecting outcomes. Our single-cell atlas (see ‘Data availability’ section) defines COVID-19-enriched neutrophil states and molecular mechanisms of dexamethasone action to develop targeted immunotherapies for severe COVID-19.

## Abstract (web summary)

New results shed light on the molecular mechanisms of dexamethasone action, underlying its therapeutic benefit in patients with severe COVID-19.

## Main

**Extended Data Fig. 1:**
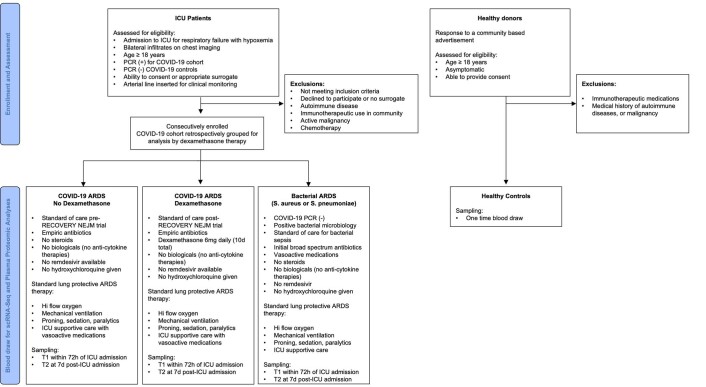
A modified CONsolidated Standards Of Reporting Trials (CONSORT) diagram showing trial groups in this study.

A broad array of viral and bacterial infections can induce diffuse lung damage, ARDS, respiratory failure and death^1–3^. Conventionally, neutrophils are thought to be key drivers of ARDS^4–6^; however, neutrophil responses during severe acute respiratory syndrome coronavirus 2 (SARS-CoV-2) are still being explored. Moreover, it is unclear if lung injury and ARDS observed in COVID-19 share common or distinct neutrophil responses and pathways of inflammation. Although recent studies have leveraged single-cell transcriptomics to dissect peripheral^7–9^ and bronchoalveolar fluid ^10–12^ immune landscapes driving COVID-19 pathogenesis, the protocols used can inadvertently exclude polymorphonuclear granulocytes, including neutrophils, as they are sensitive cells with low RNA (and high RNase) content. In this study, like others specifically investigating neutrophils^13,14^, we employed whole-blood-preserving protocols that capture neutrophils (along with all other immune cell types) from critically ill patients admitted to intensive care units (ICUs) (Extended Data Fig. 1).

Relative to bacterial ARDS, COVID-19 was associated with preferential expansion of interferon (IFN^active^) and prostaglandin (PG^active^) neutrophil states. Bacterial ARDS neutrophils had higher gene expression of anti-bacterial molecules, such as *PLAC8* and *CD83*. Although steroids remain controversial for other forms of ARDS, dexamethasone has proven to reduce mortality in severe COVID-19 (ref. ^15^). In our non-randomized, pragmatic investigation, dexamethasone in severe COVID-19 affected circulating neutrophils, altered the IFN^active^ state, downregulated interferon-responsive genes and activated IL-1R2^+^ neutrophils. Dexamethasone also induced the emergence of immature neutrophils expressing *ARG1* and *ANXA1*, genes encoding immunosuppressive molecules, which were absent in healthy controls. Additionally, dexamethasone exhibited sex-dependent effects, which might have important implications for sex-dependent outcomes and therapeutic efficacy in severe COVID-19.

## Results

### COVID-19 ARDS host responses in the context of bacterial ARDS

**Extended Data Fig. 2:**
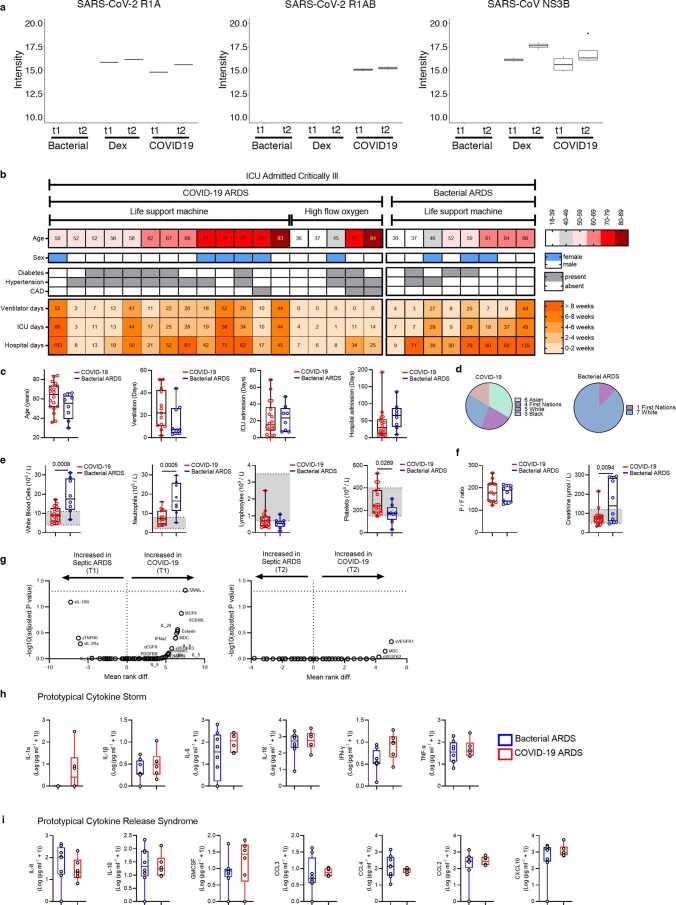
Clinical data of ICU admitted COVID-19 and bacterial ARDS. Shotgun proteomics assessment using tandem Mass Spectroscopy with a targeted search run for known SARS-CoV-2 proteins R1A and R1AB, and SARS-CoV protein NS3B are displayed for all patient cohorts (COVID-19 non-dexamethasone = 9; bacterial ARDS controls = 6). **b**. Summary of individual information of ICU admitted patients with established COVID-19 or a diagnosis of bacterial ARDS due to sepsis (bacterial ARDS n = 5 at t1, n = 4 at t2; COVID-19 ARDS n = 8 at t1, n = 4 at t2). Age, sex, comorbidities and lengths of stay are displayed. Life support machine includes mechanical ventilation (and ECMO in the instance of sample C3 at t2). **c**-**d**. Aggregated cohort clinical data (c) and racial backgrounds (d). **e**. Clinical cell counts from peripheral blood taken on t1; shaded areas show local lab normal values. **f**. PaO2/FiO2 ratio (P/F) and creatinine at t1. **g**. Multiple comparison analysis of all serum cytokines assessed at t1 and t2 are shown as volcano plots. Significance was estimated using two-tailed Mann-Whitney U test with Holm–Sidak multiple-testing correction. (**h**-**i**) Serum cytokine determination of prototypical mediators involved in (h) cytokine storm and (i) cytokine release syndrome graphed in Log transformation taken at t1. Box plots include a line across the box, upper hinge, and lower hinge which represent median, 75th percentile (Q3), and 25th percentile (Q1), respectively. The lower and upper hinges correspond to the first and third quartiles. The upper whisker extends from the hinge to the largest value no further than Q3 + 1.5× interquartile range (IQR). The lower whisker extends from the hinge to the smallest value at most Q1 – 1.5 * IQR.

Patients with life-threatening infections requiring ICU admission receive invasive procedures, medications and intense nursing care. This includes advanced invasive or non-invasive breathing support, broad-spectrum antibiotics, sedatives, narcotics, anaesthetics, paralytics, anti-coagulants, fluids and enteral nutrition. Patients require invasive lines, including central venous and arterial catheters. These interventions make it impossible to compare life-threatening infections admitted to the ICU to mild/moderate infections (treated either on the ward or in the community) or to healthy humans. To better understand COVID-19 immune response, we compared patients with COVID-19 who were admitted to the ICU to patients with life-threatening bacterial pneumonias with ARDS who were also admitted to the ICU, to account for ICU confounders. We additionally compared these groups to healthy volunteers. ICU-admitted viral ARDS (for example, H1N1) would have been an interesting comparison to contextualize COVID-19-specific response; however, eradication of flu cases globally^16^ made it infeasible. All patients with COVID-19 were assessed for bacterial infection by culture and tested negative. All patients with COVID-19 tested positive for SARS-CoV-2 by RT–PCR. We previously confirmed an absence of viral mRNA in any circulating immune cells^17^. However, plasma proteomics for SARS-CoV-2-specific viral proteins detected one or more viral proteins in all COVID-19 patient serum (Extended Data Fig. 2a and Supplementary Table 1). We first compared patients with COVID-19 ARDS to bacterial sepsis (due to respiratory *Staphylococcus* *aureus* or *Streptococcus* *pneumoniae* infection) leading to ARDS, herein referred to as bacterial ARDS (Extended Data Fig. 2b). COVID-19 ARDS donors included in this comparison did not receive dexamethasone (or other immunomodulatory agents) to capture a pharmacologically unperturbed landscape (Extended Data Fig. 1 and Supplementary Table 2). We used the modified criteria for COVID-19-associated ARDS published by the World Health Organization^18^, which include acute onset hypoxemia and bilateral pulmonary infiltrates on X-ray without evidence of cardiac failure, with a PaO_2_/FiO_2_ ratio less than 300 mmHg during mechanical ventilation or a SpO_2_/FiO2 ratio less than or equal to 315 mmHg in the absence of mechanical ventilation. Our comparison included six bacterial ARDS (*n* = 5 at time point 1 (t1) and *n* = 4 at time point 2 (t2)) and eight non-dexamethasone COVID-19 ARDS (*n* = 8 at t1 and *n* = 4 at t2) (Supplementary Table 2). Comparison of Sequential Organ Failure Assessment (SOFA) scores revealed no statistical difference in severity across COVID-19 ARDS versus bacterial ARDS (*P* = 0.17384), suggesting that these two cohorts comprised patients with similar disease severity. Bacterial ARDS was our comparator for COVID-19 ARDS because it was the closest control possible, as severe viral infections with ARDS were not accessible due to unusually low ICU admissions during the study period^19^.

Patient cohorts had similar ages, sex, days on life support and time in hospital, but patients with COVID-19 had broader racial diversity (Extended Data Fig. 2c,d and Supplementary Table 2). Bacterial ARDS induced significant neutrophilia and relative thrombocytopenia compared to near-normal circulating neutrophil numbers in COVID-19, whereas both had similar degrees of lymphopenia (Extended Data Fig. 2e). Both cohorts had similar PaO_2_/FiO_2_ ratios, an indicator of ARDS severity^20^, but patients with bacterial ARDS had significantly more kidney injury, as shown by higher serum creatinine levels (Extended Data Fig. 2f). We compared soluble inflammatory markers (Extended Data Fig. 2g) used to distinguish prototypical states, including those identified during ‘cytokine storm’ (Extended Data Fig. 2h) and ‘cytokine release syndrome’ (Extended Data Fig. 2i)^21^, which showed similar soluble cytokine and chemokine responses between infections. Therefore, life-threatening bacterial ARDS and COVID-19 ARDS had normal-to-elevated neutrophil counts, similar IL-6 levels and less organ failure as indicated by serum creatinine levels, all of which have been proposed as markers of COVID-19 severity^22,23^. This prompted nuanced investigation into immune cell states and composition.

**Fig. 1:**
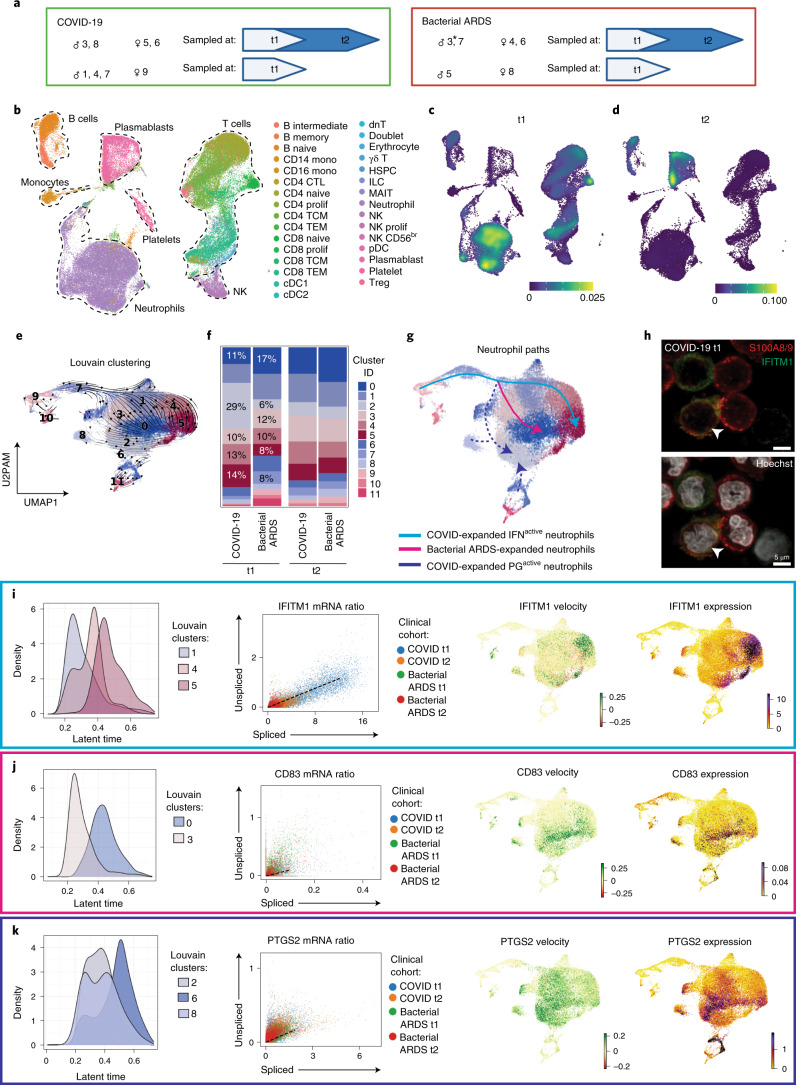
COVID-19 alters neutrophil maturation. **a**, Schematic summarizing patients with COVID-19 and bacterial ARDS profiled at t1 and t2. Comparisons presented included six bacterial ARDS (*n* = 5 at t1 and *n* = 4 at t2; * denotes that patient B3 had only the t2 sample pass QC and was not included at t1) and eight non-dexamethasone COVID-19 ARDS (*n* = 8 at t1 and *n* = 4 at t2) patients who were admitted to the ICU. **b**, UMAP projection of 86,935 whole blood cells from 21 patient samples, colored by Azimuth reference-mapped immune cell states. **c**, **d**, Kernel density estimates depicting magnitude of molecular response elicited by immune cell subsets during COVID-19 compared to bacterial ARDS at t1 (**c**) and t2 (**d**), calculated by summing DEG FCs for each cell state shown in **a**. **e**, UMAP plotting RNA velocity analysis of 29,653 subclustered neutrophils undergoing state transitions, colored by cluster ID. **f**, Stacked bar plot depicting cluster composition of clinical cohorts examined. **g**, UMAP colored by neutrophil clusters and overlaid with summary path curves based on vector fields and neutrophil state compositions in **d** and **e**, respectively, to determine neutrophil states. **h**, Immunocytochemistry for S100A8/A9 (red) and IFITM1 (green) expression on leukocyte-rich preparation from a donor with COVID-19 at t1 (representative image provided from *n* = 3 replicates). **i**–**k**, Transcriptional kinetics driving expansion of IFN^active^ (**i**), bacterial ARDS-enriched (**j**) and PG^active^ (**k**) neutrophils. Latent time distribution of trajectory-associated Louvain clusters (left), phase portraits with equilibrium slopes of spliced–unspliced ratios (center) and RNA velocity and gene expression (right) of selected genes driving divergent maturation trajectories. Phase portraits are colored by clinical cohort.

**Extended Data Fig. 3:**
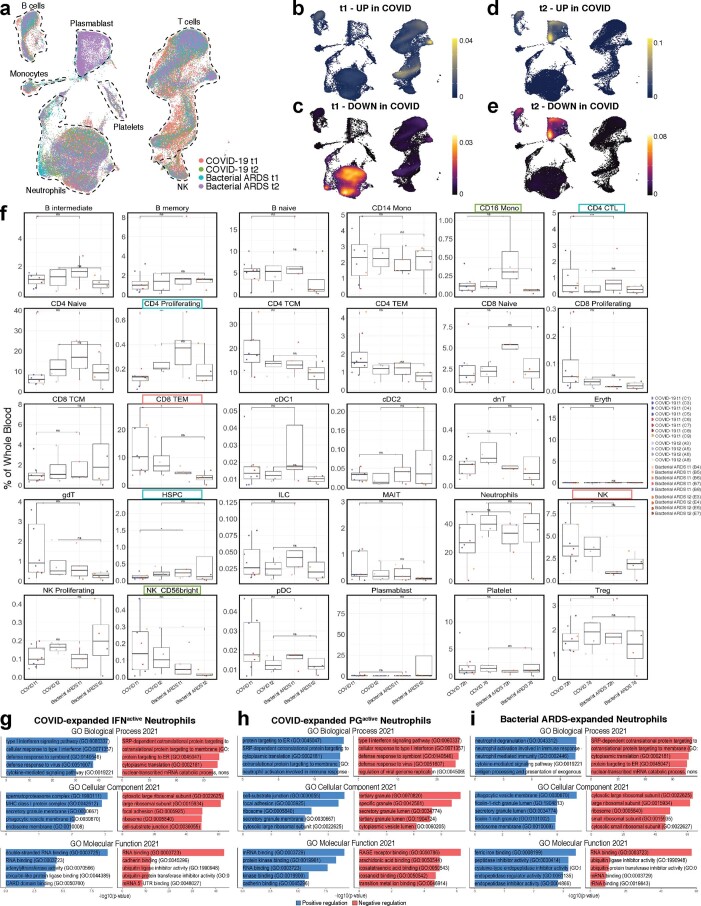
COVID-19 elicits distinct innate and adaptive immune responses compared to bacterial ARDS. **a**. UMAP projection of 86,935 whole blood cells from 21 patient samples, coloured by clinical cohort. **b**-**e**. Kernel density estimates depicting magnitude of response elicited by immune cell subsets in COVID-19 t1 (b-c) and t2 (d-e) calculated by summing consensus DEG fold changes for each cell subset shown in Panel B. Consensus DEGs upregulated in COVID-19 are plotted on cividis spectrum (yellow = higher expression) whereas downregulated DEGs are plotted on inferno spectrum (yellow/orange = lower expression). **f**. Boxplots showing percentage of each cell type in each patient sample grouped by clinical cohort and coloured by donor ID. The x axes correspond to the clinical cohort of each patient. Biologically independent samples for COVID-19 at t1 (n = 8), COVID-19 at t2 (n = 4), bacterial ARDS at t1 (n = 5), and bacterial ARDS at t2 (n = 4). Significance of effects was estimated using two-sided generalized linear mixed-effects model (glmer, binomial distribution) with fixed (COVID-19 vs Bacterial ARDS) and random (per patient) effects. P-values were calculated using likelihood-ratio chi-square for generalized linear models (using R package ‘car’) and were adjusted for multiple comparisons using Bonferroni correction. All effects with p < 0.05 are indicated. *p < 0.05, **p < 0.01, ***p < 0.001. Absolute p-adjusted values are provided in Supplementary Table 8. **g**-**i**. Gene Ontology (GO) enrichment depicting top five positively (blue) and negatively (red) regulated terms across all three domains (cellular component, biological process, and molecular function) describing biological activity of gene signatures for IFN^active^ (g), PG^active^ (h), and bacterial-expanded (i) neutrophil states.

Our queryable atlas (see ‘Data availability’ section) contains single-cell RNA sequencing (scRNA-seq) data performed on whole blood at t1 (<72 h after ICU admission) and t2 (7 d after t1) (Fig. 1a). Cellular identity was mapped to 30 immune cell types/states using uniform manifold approximation and projection (UMAP) from 21 patients and 86,935 cells (Fig. 1b and Extended Data Fig. 3a). Global magnitude of gene expression was directly compared between patients with COVID-19 and patients with bacterial ARDS (Supplementary Table 4), which revealed a more globally altered distribution of differential expression at t1 than at t2. Altered regulation of genes was most pronounced in neutrophils at t1, with lower neutrophil gene expression in COVID-19 compared to bacterial ARDS (Fig. 1c and Extended Data Fig. 3b,c). At t2, the global alterations in gene expression when comparing COVID-19 to bacterial ARDS were most pronounced in plasmablasts (Fig. 1d and Extended Data Fig. 3d,e). We quantified proportions of known peripheral blood constituents, which highlighted significant differences in CD4 T cells, CD8 T cells and natural killer (NK) cells (Extended Data Fig. 3f). These data highlight substantial global differences in immune profiles between COVID-19 and bacterial ARDS.

### COVID-19 drives enrichment of distinct neutrophil states

**Extended Data Fig. 4:**
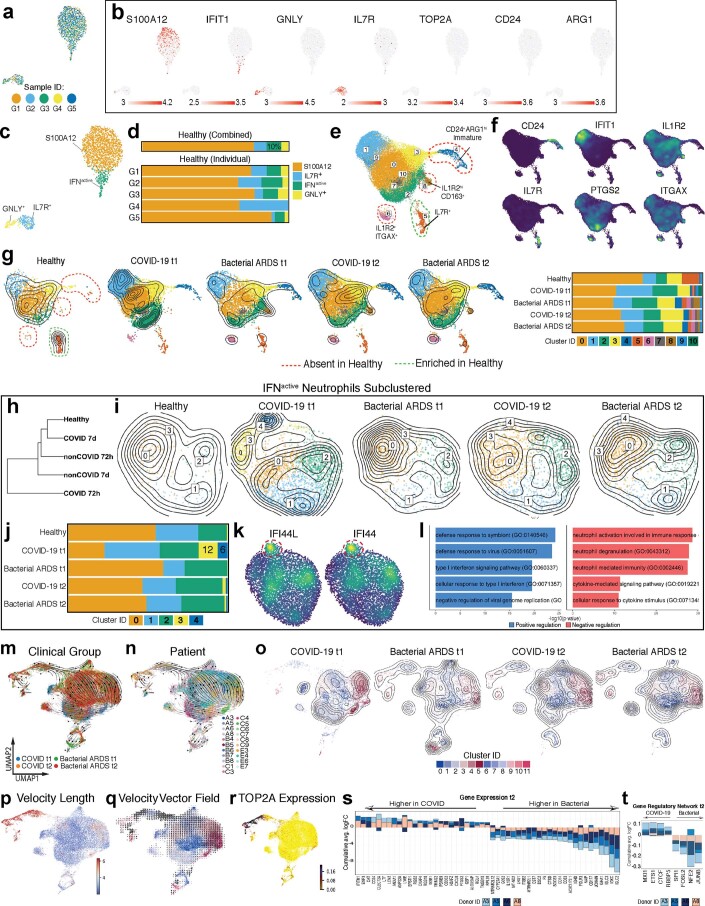
COVID-19 infection reprograms neutrophil maturation by driving expansion of ISG- and PG-expressing subsets. **a**. UMAP of neutrophils from healthy donors (n = 1,912 cells) colored by donor of origin. **b**. Neutrophil state-defining markers. **c**. UMAP of neutrophils from healthy donors colored by neutrophil states. **d**. Neutrophil state composition in healthy donors, combined across all donors or separated by individual donor ID. **e**. Subclustered neutrophils, integrated across patient cohorts to compare healthy, bacterial ARDS at t1 and t2, and COVID-19 ARDS at t1 and t2. **f**. Kernel density plots showing expression of neutrophil state-defining markers. **g**. Cohort-separated UMAPs, colored by subcluster ID and overlaid with cell density contour plots, and bar plot depicting neutrophil cluster composition across cohorts. **h**. Dendrogram showing unsupervised hierarchical clustering of IFN^active^ neutrophils by showing relatedness of this subset across patient cohorts. **i**-**j**. Cohort-separated UMAPs of IFN^active^ neutrophils, colored by subcluster ID and overlaid with cell density contour plots (i), and bar plot showing cluster composition (j) across cohorts. **k**-**l**. Expression of genes (k) and signalling pathways (l) enriched in COVID-19 t1 IFN^active^ neutrophil relative to remaining cohorts. **m**-**n**. UMAP plotting velocity analysis of 29,653 subclustered neutrophils undergoing state transitions, coloured by clinical cohort (m) and donor IDs (n). **o**. Cohort-separated UMAPs, colored by neutrophil subcluster ID and overlaid with cell density contour plots. **p**-**q**. Subclustered neutrophil UMAPs, coloured by magnitude of velocity vector length reflecting the difference between expected versus recovered unspliced counts (p) and neutrophil Louvain clusters overlaid with velocity vector fields (q). **r**. Expression of immature neutrophil marker TOP2A. s-t. Consensus plot of differentially expressed genes (s) and SCENIC-inferred transcription factors (t) upregulated (positive logFC) or suppressed (negative logFC) in neutrophils from at least 2 of 4 patients with COVID-19 relative to bacterial ARDS at t2.

Neutrophils are a primary participant in the development of ARDS^24^; yet despite similar severity of ARDS between bacterial and COVID-19 cohorts, the numbers of circulating neutrophils from clinical counts were significantly different (Extended Data Fig. 2e). Global expression differences led us to hypothesize that neutrophil qualitative states might be important determinants of disease. To interrogate neutrophil dynamics, we compared pathogen-activated neutrophils in COVID-19 and bacterial ARDS to their unperturbed counterparts in healthy donors (Extended Data Fig. 4a–l). Neutrophil subclustering and integration across healthy controls, bacterial ARDS at t1 and t2 and COVID-19 ARDS at t1 and t2 revealed an absence of immature (CD24^+^ARG1^+^) and IL-1R2^hi^ (IL-1R2^hi^CD163^+^ cluster 8 and IL-1R2^hi^ITGAX^+^ cluster 6) neutrophil states but expanded IL-7R^+^ neutrophils in healthy controls (Extended Data Fig. 4e–g). Although IFN^active^ neutrophils were conserved across healthy controls, bacterial ARDS and COVID-19 ARDS (Extended Data Fig. 4a-g), deeper subclustering of IFN^active^ neutrophils revealed an emergence of discrete substates in response to COVID-19 that were not observed in either healthy controls or bacterial ARDS (Extended Data Fig. 4h–l). These substates were enriched in interferon-induced genes *IFI44L* and *IFI44* (Extended Data Fig. 4k), molecules known to restrict respiratory viral replication^25^, and exhibited an intensified type 1 IFN activation (Extended Data Fig. 4l) relative to non-COVID-19 IFN^active^ neutrophils.

To map pathogen-activated neutrophil dynamics with high resolution, subsequent analyses employed principal components with top-loading genes that distinguish different pathogen-activated states arising during COVID-19 and bacterial ARDS (and not healthy controls) for downstream dimensionality reduction. Neutrophils were subjected to velocity analysis^26,27^ to reconstruct maturation dynamics. Louvain clusters (Fig. 1e), clinical cohorts, individual patients and velocity length were overlayed on velocity vector fields (Extended Data Fig. 4m–q), showing three main neutrophil states. The proportions of neutrophil states were compared at t1, and this revealed a divergent expansion of IFN^active^ neutrophils (clusters 2, 4 and 5) marked by *IFITM1* expression in COVID-19, which became similar to bacterial ARDS at t2 (Fig. 1f–h and Extended Data Fig. 4m–o). Gene expression of *IFITM1* in neutrophils from patients with COVID-19 at t1 was confirmed by immunofluorescent staining for IFITM1 protein, co-localized with S100A8/9, and typical neutrophil nuclear morphology (Fig. 1h and Extended Data Fig. 10).

Classically, peripheral neutrophils are considered non-dividing and terminally differentiated; however, the increase in velocity length suggested the ability to alter phenotypic states once in circulation along specific paths or ‘lineages’. COVID-19 neutrophils followed unique maturation paths compared to bacterial ARDS, culminating in three distinct terminal states: IFN^active^, PG^active^ or bacterial ARDS enriched (Fig. 1e–g and Extended Data Fig. 4m–o). The apex of this trajectory was marked by high velocity lengths, characteristic of cells undergoing differentiation (Extended Data Fig. 4p,q). COVID-19 neutrophils preferentially transitioned from the apex of the trajectory, which was an immature state (*TOP2A-*expressing; Extended Data Fig. 4r) to an IFN^active^ state characterized by *IFITM1*, *IFITM2* and *IFI6* expression (clusters 1–4 and 5; Fig. 1i; Online Atlas) and activation of type I IFN signaling pathways (Extended Data Fig. 3g). Topological and geometry features of the neutrophil vector field, including identification of attractor and saddle points, were solved in an unsupervised fashion using the vector field function in Dynamo^28^. The continuum of neutrophil states culminating in stable IFN^active^ and bacterial-enriched states, as well as unstable PG^active^ attractor state, is shown in Supplementary Video 1. Lineage relationship was less clear for COVID-19-enriched PG^active^ clusters defined by PG responsive genes (clusters 2, 6 and 8), with notable increases in *PTGER4* and *PTGS2* (or COX2), which encode a proposed target in COVID-19 (ref. ^29^) (Fig. 1k and Extended Data Fig. 4s,t; Online Atlas). PG^active^ neutrophils exhibited relative enrichment in adhesive capacity suggested by overrepresentation of cell–matrix junction pathways, such as focal adhesions mediated by *TLN1*, *ADAM10*, *RHOB*, *CD46* and *ADGRE5* (CD97), which encodes a mechanosensitive G-protein-coupled receptor (Extended Data Fig. 3h). The dominant bacterial ARDS state was characterized by expression of genes that encode anti-bacterial proteins CD83 (ref. ^30^), CD177 and PLAC8 (ref. ^31^) (clusters 3–0; Fig. 1j; Online Atlas). Interestingly, bacterial-enriched neutrophils were predicted to harbor ficolin-1-rich granules (Extended Data Fig. 3i). Because ficolin-1 is a recognition molecule that binds to carbohydrate structures in bacteria to initiate lectin complement pathway^32^, its enrichment suggests a poised state for targeting a broad range of bacterial pathogens. Together, these data showed that peripheral neutrophils have dynamic programming abilities that result in neutrophil polarization defined by emergence of IFN^active^ and PG^active^ neutrophil states in severe COVID-19.

### Unique regulatory pathways control neutrophil maturation

**Fig. 2:**
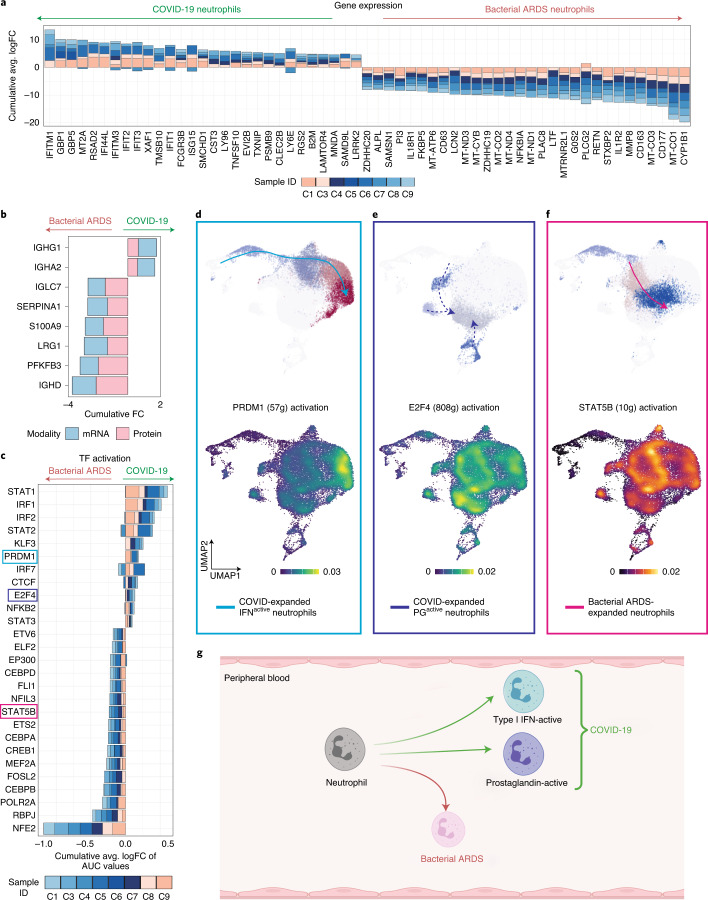
Distinct regulatory programs drive divergent neutrophil maturation. **a**, Consensus neutrophil DEGs upregulated (positive FC) or suppressed (negative FC) during COVID-19 in at least three of eight patients at t1 relative to bacterial ARDS. None of the patients with COVID-19 ARDS included in this comparison received dexamethasone. **b**, Consensus of differentially expressed features distinguishing neutrophils in COVID-19 versus bacterial ARDS jointly identified by changes in mRNA (quantified by scRNA-seq) and plasma protein (quantified by LC–MS/MS) levels. **c**, Differentially activated consensus TFs in neutrophils from patients with COVID-19 relative to bacterial ARDS at t1. Stacked bars depict logFC contributions of each patient with COVID-19. **d**–**f**, Gene regulatory networks preferentially driving IFN^active^ (PRDM1, **d**), PG^active^ (E2F4, **e**) and bacterial ARDS-enriched (STAT5B, **f**) neutrophil states. Scale bars depict kernel density estimates approximating magnitude of TF activation inferred by SCENIC-calculated AUCell scores. **g**, Schematic summarizing neutrophil fates favored during COVID-19 versus bacterial ARDS (created with BioRender).

Rapid and robust IFN responses protect against COVID-19 severe disease, whereas delayed responses could exacerbate systemic and pulmonary inflammation^33,34^. Neutrophil IFN responses are not traditionally considered during infections, and neutrophils are generally considered to be homogenous, with a uniform pro-inflammatory capacity. Global neutrophil expression aligned with neutrophil state-specific markers, such as interferon response genes (*IFITM1*, *RSAD2*, *IFI6* and *ISG10*), being more highly expressed in COVID-19 neutrophils (Fig. 2a and Extended Data Fig. 4f). The inverse was the case for genes encoding anti-bacterial proteins, such as *PLAC8* (Fig. 2a; Online Atlas). To interrogate infection-specific neutrophil response, we shortlisted differentially expressed features identified jointly by concordant gene and plasma protein expression changes (Fig. 2b). Interestingly, *SERPINA1* (encoding protease inhibitor α-1 antitrypsin) and *PFKFB3* (encoding phosphofructokinase, a key regulator of glycolysis) were suppressed in COVID-19 neutrophils, suggesting divergence in granule-associated enzyme composition and metabolic states. Identification of differential neutrophil states prompted further exploration into factors driving neutrophil state polarization. Gene regulatory network reconstruction using single-cell regulatory network inference and clustering (SCENIC)^35^ revealed differentially activated transcription factors (TFs) STAT1, IRF2 and PRDM1 in COVID-19 (Fig. 2c), whereas bacterial ARDS neutrophils had increased prototypical granulocyte TFs, such as CEBPA, CEBPB and STAT5B, and less defined factors such as NFE2 (Fig. 2c; Online Atlas). PRDM1 activation was most pronounced in the IFN^active^ neutrophil population and was likely responsible for driving expression of IFN response elements (*IFIT1*, *ISG15* and *IFI6*) and anti-viral signaling, such as *RSAD2* and *STAT1* (Fig. 2d and Supplementary Table 7; Online Atlas). A hallmark of PG^active^ neutrophil polarization was activation of E2F4, predicted to drive 808 genes (Fig. 2e and Supplementary Table 7), whereas neutrophil programming during bacterial ARDS included activation of STAT5B that was predicted to be upstream of ten genes (Fig. 2f and Supplementary Table 7). Consistent with the role of E2F4 as a transcriptional repressor mediating cell cycle arrest^36^, negative regulation of cell cycle progression was an overrepresented pathway in its SCENIC-inferred targetome (Supplementary Table 7). Interestingly, a subset of the E2F4 targetome was associated with regulating assembly of cell–matrix junctions (Supplementary Table 7), corroborating the relative enrichment in adhesive capacity seen within PG^active^ neutrophils (Extended Data Fig. 3h). To summarize, in response to COVID-19, neutrophils were polarized by unique transcriptional regulation toward one of two main populations: either an IFN^active^ population or a PG^active^ population (Fig. 2g).

### Dexamethasone alters immune dynamics and plasma proteomics

**Fig. 3:**
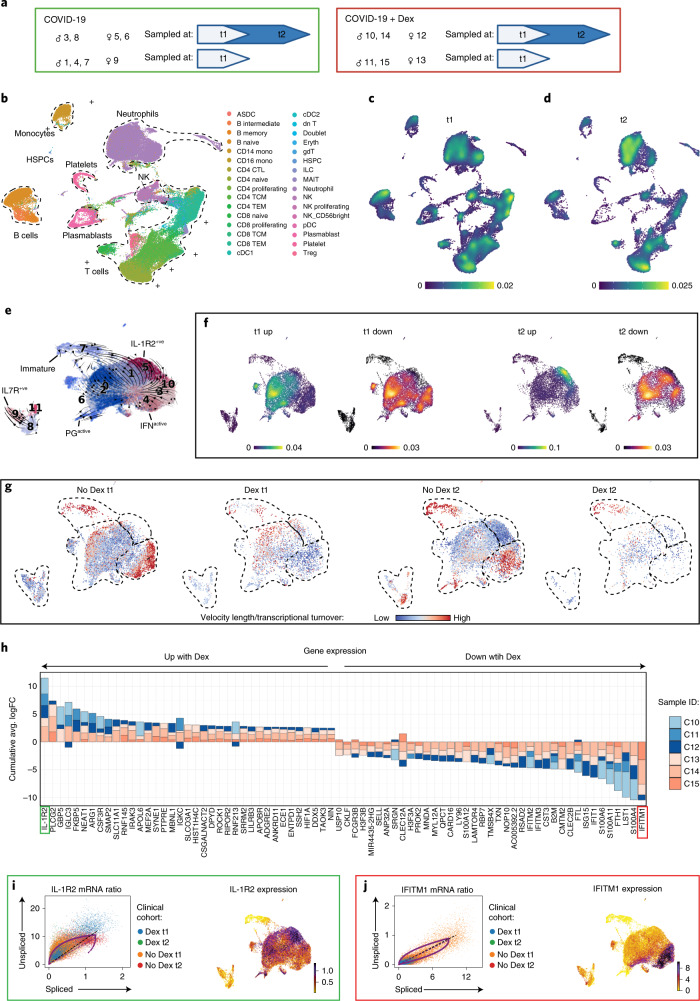
Dexamethasone suppresses IFN programs and depletes IFN^active^ neutrophils in COVID-19. **a**, Schematic summarizing patients with COVID-19 who were treated with or without dexamethasone profiled at t1 and t2. Comparisons presented included eight non-dexamethasone-treated COVID-19 ARDS (*n* = 8 at t1 and *n* = 4 at t2) and six dexamethasone-treated COVID-19 ARDS (*n* = 6 at t1 and *n* = 3 at t2) patients who were admitted to the ICU. **b**, UMAP projection of 80,994 whole blood cells from 21 patient samples, colored by Azimuth reference-mapped immune cell states. **c**, **d**, Kernel density estimates depicting magnitude of molecular response elicited by immune cell subsets after dexamethasone treatment at t1 (**c**) and t2 (**d**), calculated by summing DEG FCs for each cell state shown in **a**. **e**, Neutrophil states overlaid on a UMAP of 23,193 subclustered neutrophils from dexamethasone- and non-dexamethasone-treated patients with COVID-19, colored by cluster ID. **f**, Magnitude of molecular response elicited by each neutrophil state after dexamethasone treatment calculated by summing DEG FCs for each cell state shown in **d**, **g**, RNA velocity vector length (indicating rate of differentiation/state transition) in dexamethasone- and non-dexamethasone-treated neutrophils at t1 and t2. **h**, Consensus neutrophil DEGs upregulated (positive FC) or suppressed (negative FC) after dexamethasone in at least three of six patients with COVID-19 at t1 relative to non-dexamethasone COVID-19 controls. Stacked bars depict logFC contribution of each dexamethasone-treated patient. **i**, **j**, Differential splicing kinetics drives activation of IL-1R2 (**i**) and suppression of IFITM1 expression (**j**) after dexamethasone treatment. Phase portraits show equilibrium slopes of spliced–unspliced mRNA ratios. Green denotes most upregulated and red denotes most downregulated DEGs with COVID-19 (**f**). HSPC, hematopoietic stem and progenitor cell. Dex, dexamethasone.

**Extended Data Fig. 5:**
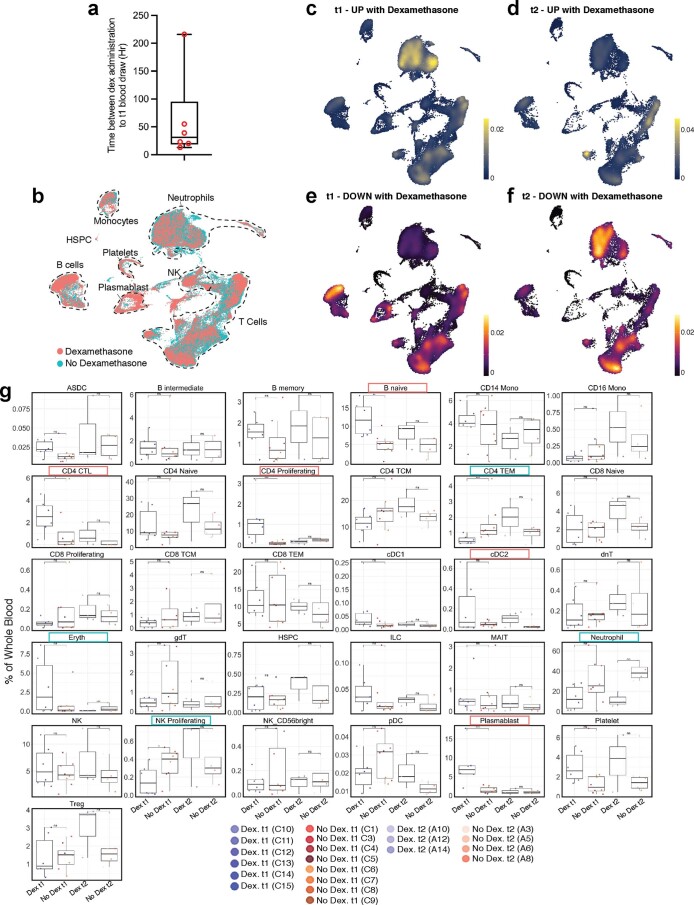
Immunosuppressive effects of dexamethasone are mediated through neutrophils and multiple adaptive immune cell subsets. **a**. Bar plot shows distribution of time interval (in hours) between dexamethasone administration to first blood draw at t1. Box plot include a line across the box, upper hinge, and lower hinge which represent median, Q3, Q1, respectively. The lower and upper hinges correspond to the Q1 and Q3. The upper whisker extends from the hinge to the largest value no further than Q3 + 1.5× interquartile range (IQR). The lower whisker extends from the hinge to the smallest value at most Q1 – 1.5 * IQR. **b**. UMAP projection of 80,994 whole blood cells from 21 patient samples, coloured by treatment groups (non-dexamethasone COVID-19 ARDS (n = 8 at t1, n = 4 at t2; dexamethasone-treated COVID-19 ARDS (n = 6 at t1, n = 3 at t2)) **c**-**f**. Kernel density estimates depicting magnitude of response elicited by immune cell subsets following dexamethasone treatment at 72 hours post-ICU (c-d) and 7 days post-ICU (e-f) calculated by summing consensus DEG fold changes for each cell subset shown in Panel B. Consensus DEGs upregulated following dexamethasone treatment are plotted on cividis spectrum whereas downregulated DEGs are plotted on inferno spectrum. **g**. Boxplots showing percentage of each cell type in each patient sample grouped by treatment and coloured by donor ID. The x axes correspond to four treatment groups. n = 6, n = 8, n = 3, and n = 4 biologically independent samples from dexamethasone-treated t1, no dexamethasone t1, dexamethasone-treated t2, no dexamethasone t2, respectively. Significance of effects was estimated using two-sided generalized linear mixed-effects model (glmer, binomial distribution) with fixed (dexamethasone vs no dexamethasone) and random (per patient) effects. P-values were calculated using likelihood-ratio chi-square for generalized linear models (using R package ‘car’) and were adjusted for multiple comparisons using Bonferroni correction. All effects with p < 0.05 are indicated. *p < 0.05, **p < 0.01, ***p < 0.001. Absolute p-adjusted values are provided in Supplementary Table 8.

Conventional therapeutics have limited efficacy for COVID-19, and, although dexamethasone offers a moderate benefit, the RECOVERY trial reported that the benefit was greatest in the most severely affected patients^15^. Mechanisms underlying this benefit are unclear and not universal, so opportunity exists to optimize or better target this therapy. In this study, we compared eight non-dexamethasone COVID-19 ARDS (*n* = 8 at t1 and *n* = 4 at t2) to six dexamethasone-treated COVID-19 ARDS (*n* = 6 at t1 and *n* = 3 at t2) patients admitted to the ICU (Supplementary Table 2). Comparison of illness severity between non-dexamethasone- versus dexamethasone-treated patients with COVID-19 ARDS using SOFA scores obtained during ICU admission revealed no statistical difference (*P* = 0.33204), suggesting similar severity between the two groups. Median time between dexamethasone administration to t1 blood draw (within 72 h of ICU admission) was 31 h (Fig. 3a, Extended Data Fig. 5a and Supplementary Table 1). Global differences in transcription were apparent at t1, with clear upregulation of genes in neutrophils and some T cell subsets in patients with COVID-19 who were treated with dexamethasone versus those who were not treated (Fig. 3b–d, Extended Data Fig. 5b and Supplementary Table 5). At t1, the dexamethasone-treated group had globally downregulated genes in naive B cells, plasmablasts and some T cells (Extended Data Fig. 5b–d). At t2, gene upregulation occurred in adaptive immune cells, including naive and effector CD8 T cells, with limited alterations in innate myeloid lineages, including neutrophils. Neutrophils showed clear downregulation of genes at t2, as did CD4 naive and central memory T cells (Extended Data Fig. 5e,f). Proportionally, at t1, dexamethasone administration was associated with an increase in cytotoxic CD4 T cells, naive B cells and plasmablasts and decreased proliferating NK cells and CD4 effector memory cells (Extended Data Fig. 5g). By t2, dexamethasone was associated with suppressed neutrophil proportions in circulation compared to untreated COVID-19 controls (13% versus 41%; Extended Data Fig. 5g). Plasma proteomics from the same cohort revealed that dexamethasone suppressed ten host proteins (S100A8, S100A9, SERPINA1, SERPINA3, ORM1, LBP, VWF, PIGR, AZGP1 and CRP) that others have identified as biomarkers distinguishing severe COVID-19 cases from mild to moderate counterparts (full host proteome queryable via Online Atlas; Supplementary Table 3)^37–40^. Suppression of calprotectin (S100A8/S100A9) and neutrophil serine proteases (SERPINA1 and SERPINA3) in plasma, paired with depletion of neutrophil proportions, implicates the modulation of neutrophil-related inflammatory processes as a method of action for dexamethasone.

### Neutrophil IFN programs are restrained with dexamethasone

**Fig. 4:**
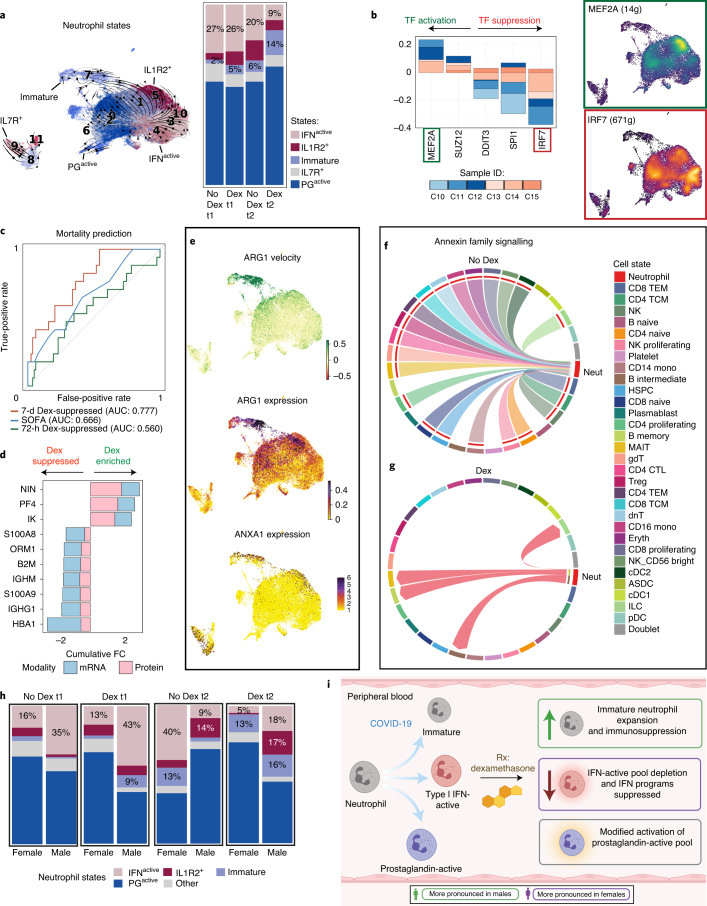
Dexamethasone expands immunosuppressive neutrophils and repatterns their interactions in COVID-19. **a**, Neutrophil states mapped onto Louvain-clustered UMAP, with comparison of neutrophil composition between dexamethasone- and non-dexamethasone-treated samples at t1 and t2. **b**, Consensus TFs activated or suppressed after dexamethasone in at least three of six patients at t1 and predicted activity of MEF2A and IRF7, two of the most differentially regulated TFs, after dexamethasone. **c**. ROC curves assessing the discriminatory capacity of dexamethasone-suppressed DEGs at t1 and t2 and SOFA scores for predicting 28-d mortality in a validation cohort of 103 bulk whole blood RNA-seq samples where 17 cases were fatal. **d**, Consensus of differentially expressed neutrophil features upregulated (positive FC) or suppressed (negative FC) after dexamethasone jointly identified by changes in mRNA (quantified by scRNA-seq) and plasma protein (quantified by LC–MS/MS) levels. **e**, Immature and IL-1R2^+^ neutrophil subsets express high levels of immunosuppressive neutrophil markers ARG1 and ANXA1. **f**, **g**, Topology of annexin signaling family without (**f**) and with (**g**) dexamethasone treatment (edges filtered to those where neutrophils function as senders or recipients of annexin signals). **h**, Neutrophil state composition separated by sex and dexamethasone status at t1 and t2. **i**, Schematic summarizing the effects of dexamethasone on neutrophil fates and function in COVID-19 after dexamethasone treatment (created with BioRender). Dex, dexamethasone.

**Extended Data Fig. 6:**
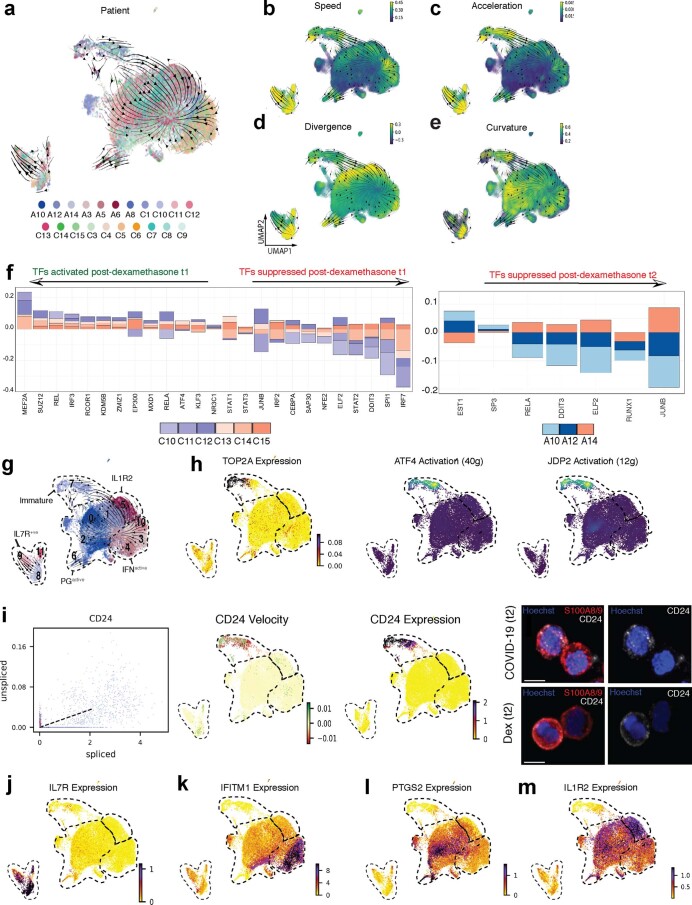
Distinct neutrophil states and their response to dexamethasone. **a**. UMAP projection of subclustered neutrophils from 21 patient samples, coloured by individual patient ID. b-e. Cell speed (length of velocity vectors; **b**), acceleration (subspaces where velocity undergoes dramatic changes in either in magnitude or direction; **c**), divergence (outward flux indicating the extent to which a point behaves like a source; **d**) and curvature (hotspots of abrupt vector field change; **e**). **f**. Differentially activated consensus TFs upregulated (positive logFC) or suppressed (negative logFC) post-dexamethasone in at least 3 of 6 patients at t1, and in at least 2 of 3 patients at t2. g-j. Neutrophil states (**g**) can be distinguished by expression of proliferative marker TOP2A and activation of immaturity-associated TFs ATF4 and JDP2 (**h**), CD24 splicing kinetics, velocity, expression and immunocytochemistry (representative images are shown; n = 3 for each group; Scale bar, 5 μm) (**i**), IL-7R (**j**), Interferon-stimulated genes such as IFITM1 (**k**), genes involved in prostaglandin synthesis such as PTGS2 (**l**), and IL-1R2 (**m**).

**Extended Data Fig. 7:**
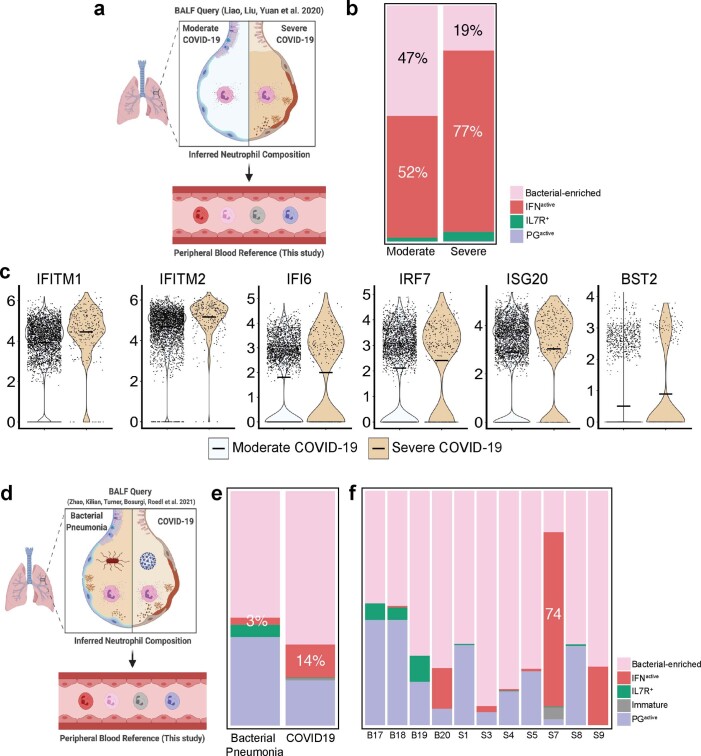
Inferred neutrophil composition in bronchoalveolar microenvironments. **a**. Strategy for inferring BALF neutrophil composition in severe and moderate COVID-19 by reference-projecting to neutrophil states in peripheral blood. **b**. Proportion of neutrophil states in bronchoalveolar microenvironment in severe and moderate COVID-19. **c**. Expression of Type 1 IFN genes in neutrophils across severe and moderate COVID-19 patients. **d**. Strategy for inferring BALF neutrophil composition in bacterial pneumonia versus COVID-19. **e**-**f**. Proportion of neutrophil states in bronchoalveolar microenvironment, separated by bacterial pneumonia and COVID-19 (e) and individual donors (f).

Owing to the early and sustained differences in neutrophil transcriptional programs, as well as their global depletion by t2 with dexamethasone, more granular effects of dexamethasone on neutrophil states were evaluated. Neutrophil reclustering again identified immature neutrophils at the apex of the maturation trajectory, accelerating and exhibiting maximal divergence before PG^active^ and IFN^active^ state commitments (Fig. 3d and Extended Data Fig. 6a–e). Interestingly, we also identified IL-7R^+^ neutrophils (comprising roughly 8% of total neutrophils) whose trajectories remained separate (Fig. 3d and Extended Data Fig. 6g,j), suggesting an entirely distinct neutrophil state. Initially, dexamethasone-treated samples had higher global transcription in PG^active^ neutrophils, whereas PG^active^ neutrophils emerged concomitant with high *IL1R2* expression (IL-1R2^+^) (Fig. 3e) at t2. Conversely, dexamethasone appeared to attenuate global transcription of IFN^active^ neutrophils at t1 and t2 (Fig. 3e,f). Remarkably, at t1 with dexamethasone dynamic state changes in IFN^active^ and IL-7R^+^, neutrophils were halted, followed by preferential depletion of IFN^active^ subsets (Fig. 3g). Indeed, although dexamethasone was associated with a reduction in global neutrophil numbers, we also detected a reduction specifically in IFN^active^ neutrophils to a proportion similar to that detected in healthy controls (9% after dexamethasone at t2 versus 10% in healthy controls) (Fig. 4a and Extended Data Fig. 4a–d). Although collection of airway samples (that is, bronchoalveolar lavage fluid (BALF)) was not feasible at our institution, we leveraged two recent BALF scRNA-seq datasets^11,41^ to assess whether IFN^active^ neutrophils dominate the bronchoalveolar landscape during severe COVID-19. Projection of CSF3R^+^S100A8^+^S100A9^+^ BALF neutrophils onto our reference revealed (1) 1.5 fold change (FC) expansion of IFN^active^ neutrophils in severe COVID-19 relative to moderate disease (77% versus 52%; Extended Data Fig. 7a,b); (2) preferential activation of IFN-stimulated genes (ISGs), such as *IFITM1*, *IFITM2*, *IFI6*, *IRF7* and *ISG20*, in severe COVID-19 neutrophils (Extended Data Fig. 7c); and (3) 4.7 FC higher IFN^active^ neutrophils in COVID-19 relative to bacterial pneumonia (14% versus 3%; Extended Data Fig. 7d–f). Albeit anecdotal, in our whole blood cohort, the IFN^active^ neutrophil state was dominant in patient S7 (ref. ^41^), an 80-year-old male with remarkably high viral titers who died from COVID-19 complications within 3–4 d of sampling (Extended Data Fig. 7f).

Consensus differentially expressed gene (DEG) analysis highlighted upregulation of *IL1R2*, which encodes a decoy receptor that sequesters IL-1, and downregulation of *IFITM1* as the most prominent discriminating features of dexamethasone treatment (Fig. 3h). Additionally, dexamethasone attenuated neutrophil expression of IFN pathways more broadly, including the reduction of *IFITM1-IFITM3*, *IFIT1*, *ISG15* and *RSAD2* (Fig. 3h). Examination of unspliced pre-mRNA to mature spliced mRNA ratios supported the notion that induction of immunoregulatory systems (that is, *IL1R2*; Fig. 3i) and suppression of IFN (that is, *IFITM1*) (Fig. 3j) programs were driven by differential upstream regulation of these pathways.

### Dexamethasone renders neutrophils more immunosuppressive

**Extended Data Fig. 8:**
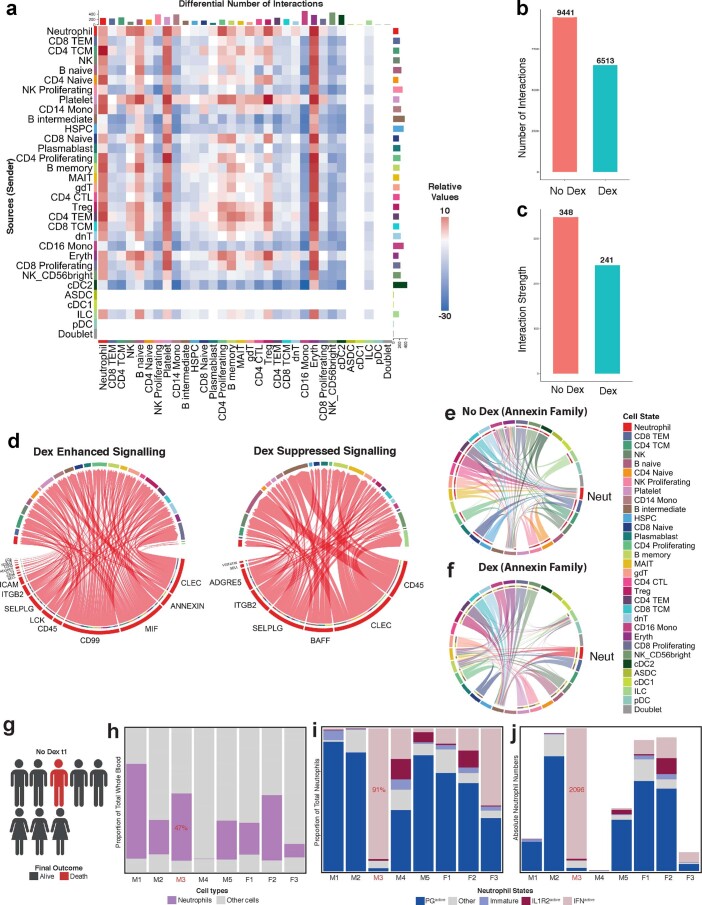
Dexamethasone alters global signaling topology and increased proportion of IFN^active^ neutrophils are associated with mortality. **a**. Interaction heatmap summarizing differential number of incoming (top bar plot) and outgoing (right bar plot) cell-to-cell interactions following dexamethasone treatment. **b**-**c**. Global summary of number (b) and strength (c) of all interactions different immune cell types with and without dexamethasone. **d**. Neutrophil-driven signaling pathways enhanced and supressed with dexamethasone, identified using CellChat (MHC-I signaling filtered out). **e**-**f**. Unfiltered topology of annexin signaling without (e) and with dexamethasone (f) treatment. **g**. Schematic depicting outcomes in non-dexamethasone treated COVID-19 patients. Male 3 (M3) succumbed to disease. **h**-**i**. Proportion of neutrophils (h) and neutrophil states (i) in whole blood samples from individual donors (5 males, 3 females) at t1. **j**. Raw neutrophil state counts from the same 8 donors at t1.

Patients treated with dexamethasone had shifted neutrophil state compositions. Although IFN^active^ neutrophils were significantly depleted at t2, there was two-fold expansion in immature neutrophils relative to non-treated COVID-19 controls (Fig. 4a and Extended Data Figs. 6h,i and 10), which was absent in the healthy controls. Albeit circumstantial, the dominance of IFN^active^ neutrophils at t1 in the patient who died from COVID-19 in the non-dexamethasone cohort suggests the possibility that depletion of IFN^active^ neutrophils might be a mechanism by which dexamethasone is protective (Extended Data Fig. 8g–j). Assessment of gene regulatory networks showed that IRF7 and MEF2A exhibited opposing activation patterns, with IRF7 being the most suppressed and MEF2A being the most enhanced TFs identified with dexamethasone, which correlates with the emergence of PG^active^ and IL-1R2^+^ states and attenuation of the IFN^active^ neutrophil states (Fig. 4b and Extended Data Fig. 6k–m). To assess the generalizability of the dexamethasone-regulated DEGs identified in our cohort, we asked whether they accurately predicted mortality due to COVID-19 in a larger validation cohort. By leveraging a whole blood bulk RNA-seq dataset from 103 patients with COVID-19 (refs. ^42,43^), we scored each sample by the aggregated expression of dexamethasone-suppressed DEGs at t1 and t2 (Supplementary Table 5). Interestingly, suppressed DEGs at t2 (but not t1) proved to be a far superior predictor of 28-d mortality (area under the curve (AUC) = 0.78; confidence interval (CI), 0.67–0.89) compared to clinical severity scales, such as SOFA (AUC = 0.67; CI, 0.51–0.82) across all classification thresholds (Fig. 4c). Multi-modal (transcriptional and plasma proteomic) assessments corroborated suppression of mature neutrophil programs (for example, β-2-microglobulin encoded by *B2M*; Online Atlas) along with concomitant activation of IFN-restraining cytokines (for example, IK cytokine, a potent inhibitor of IFN-γ) after dexamethasone (Fig. 4d). Unexpectedly, steroid administration was associated with an increase in circulating immature neutrophils, which highly expressed *TOP2A*, and activated ATF4 and JDP2, TFs seen in undifferentiated cells or those undergoing nuclear reprogramming (Extended Data Fig. 6h). Because *TOP2A* marks proliferating cells^44^, we asked whether dexamethasone increased proliferation of immature neutrophils to drive their expansion. There was no change in frequency of TOP2A^+^ immature neutrophil after dexamethasone treatment (8% TOP2A^+^ in dexamethasone-treated versus 10% in non-dexamethasone-treated across t1 and t2; *χ*^2^ = 4.58, *P* = 0.21), suggesting that dexamethasone does not stimulate division of circulating (immature) neutrophils. Immature neutrophils expressed high levels of *ARG1*, *ANXA1* (Fig. 4e) and *CD24* (both mRNA and protein; Extended Data Fig. 6i), suggesting that additional immunomodulatory function^45–49^ expanded with dexamethasone. Both *ARG1* and *ANXA1* express glucocorticoid response elements, emphasizing the possibility of their direct regulation by dexamethasone treatment^50,51^.

To further understand the role of neutrophils during COVID-19 and the effects of dexamethasone, we investigated cellular connectomes. Cellular interactions between many cell types (including highly interactive neutrophils) were noted (Extended Data Fig. 8a), and dexamethasone altered the globally predicted interactions by suppressing both number and strength of intercellular interactions (Extended Data Fig. 8b,c). Dexamethasone enhanced and suppressed (Extended Data Fig. 8d) several unique neutrophil-driven signaling networks. Annexin signaling, which was enhanced in the immature neutrophils and which are powerful glucocorticoid targets for resolving inflammation^52^, was augmented between neutrophils and the other circulating immune cells when patients received dexamethasone (Fig. 4f,g). Of note is the direction of annexin family signaling, which switched from incoming toward neutrophils to being outgoing from neutrophils toward B intermediate and memory cells and MAIT cells after dexamethasone (Fig. 4f,g and Extended Data Fig. 8e,f). Re-patterning in annexin signaling was primarily driven by a 2.4-fold expansion of immature neutrophils after dexamethasone and not due to a change in *ANXA1* expression (12.4 versus 12.0 log-normalized unique molecular identifier (UMI) in non-treated and treated, respectively) in immature subsets across non-dexamethasone-treated and dexamethasone-treated donors. Dexamethasone altered neutrophil states by promoting expansion of an ARG1^+^ANXA1^+^ immature state with immunosuppressive features and altered global communication structure such that neutrophils became active instructors of peripheral immune cells.

### Neutrophil response to dexamethasone is sexually dimorphic

**Extended Data Fig. 9:**
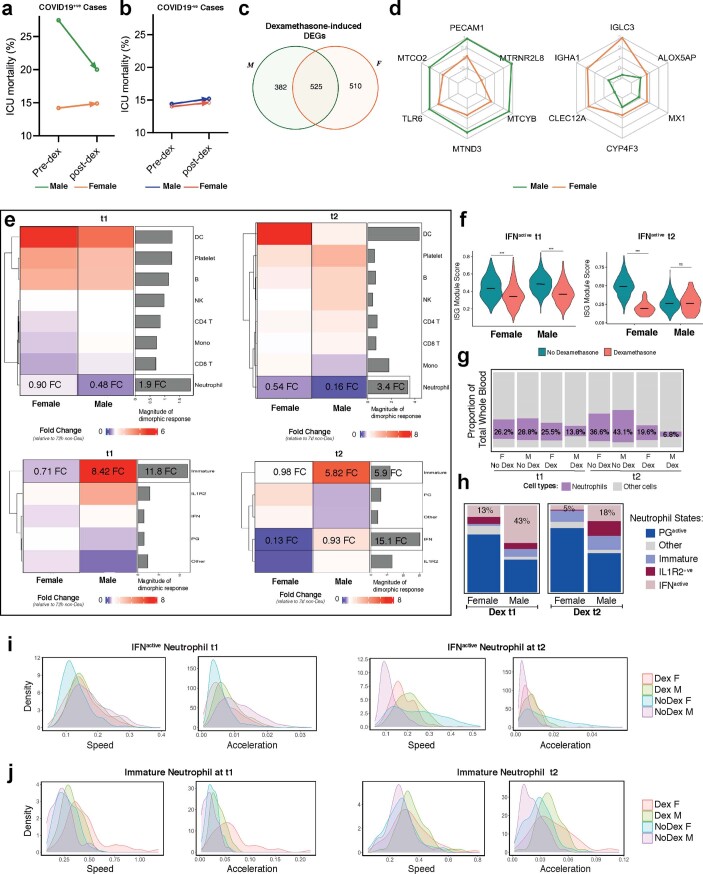
Dexamethasone attenuates neutrophil response in a sexually dimorphic fashion. **a**-**b**. ICU mortality rates of sex-separated patients with (a) or without COVID-19 (b) comparing pre-dexamethasone (January 2020 till May 31^st^, 2020) and post-dexamethasone (June 1^st^, 2020, till May 31^st^, 2021) standard of care time periods. **c**. Number of genes that are uniquely or jointly regulated with dexamethasone between males and females. **d**. Differential magnitude or direction of regulation within dexamethasone-induced DEGs jointly regulated by both sexes. **e**. Heatmap depicting dexamethasone-induced shifts in cellular composition at t1 and t2 and accompanying bar plots showing magnitude of divergence between male and female response. Dexamethasone-induced shifts in neutrophil state composition at t1 and t2 along with magnitude of divergence between male and female response. **f**. Module score of ISG signatures in ISG-active neutrophils across sex and dexamethasone treatment at t1 and t2. Statistical significance was assessed using an ANOVA test followed by bonferroni-corrected two‐sided pair-wise t-tests. * p-value < 0.05; ** p-value < 0.01; *** p-value < 0.001; ns p-value > 0.05. Absolute p-adjusted values are provided in Supplementary Table 8. Center line indicates median data point. **g**. Comparison of proportion of neutrophils in whole blood samples from sex-separated cohorts. **h**. Comparison of neutrophil composition across sex in dexamethasone-treated patients at 72 hours and 7 days post-ICU admission. **i**-**j**. Histograms depicting dynamo-calculated distribution of cell speed (length of velocity vectors) and acceleration (subspaces where velocity undergoes dramatic changes in magnitude or direction) of all IFN-active (i) and immature (j) neutrophils, separated by sex and dexamethasone treatment for both t1 and t2.

**Extended Data Fig. 10:**
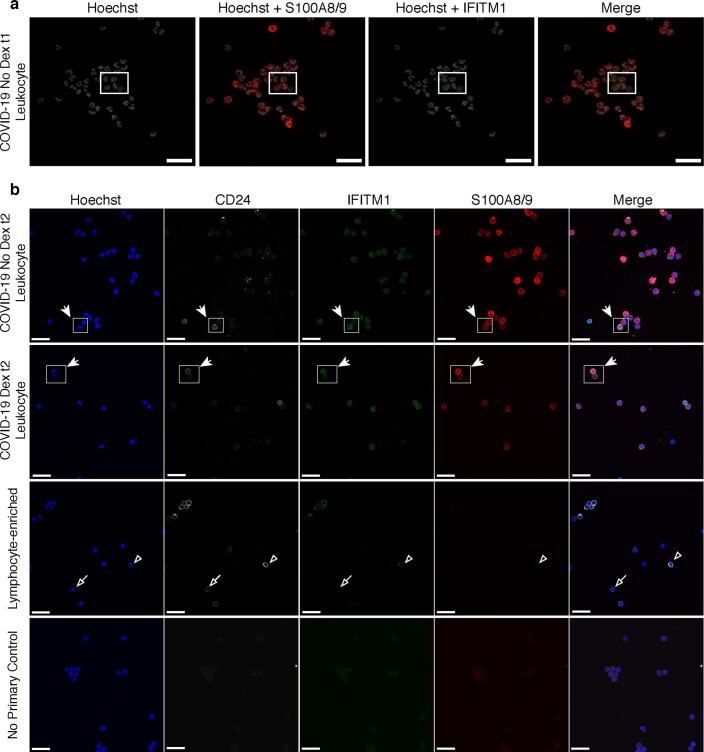
Immunofluorescence staining for neutrophil population markers. Immunofluorescence representative images showing co-staining of Hoechst dye, anti-calprotectin (S100A8/A9), anti-IFITM1, and anti-CD24 antibodies on either leukocyte- or lymphocyte-enriched cytospin preparations from COVID-19^+ve^ patients at t1 (**a**) or t2 (**b**). Rectangles highlight field of view shown in Fig. 1h (a) and Extended Data Fig. 6i (**b**). Representative images are shown; n = 3 for each group; Scale bar, 25 μm.

Given that the clinical benefit of dexamethasone is more evident in males^15^, and because males are predisposed to more severe COVID-19 presentations and outcomes^53^, we surmised that dexamethasone incites sexually dimorphic effects. Our retrospective province-wide audit comparing 72 pre-dexamethasone (51 male and 21 female) versus 1,581 post-dexamethasone (1,013 male and 568 female) ICU-admitted patients confirmed a preferential mortality benefit in male patients with COVID-19 (Extended Data Fig. 9a,b). Dexamethasone-treated patients had 525 neutrophil DEGs across both sexes, whereas 892 were uniquely modulated in either males or females (Supplementary Table 6). Of the jointly modulated DEGs, a subset (24 of 525) exhibited statistically significant dimorphism in magnitude or direction of regulation (Extended Data Fig. 9c,d). Although neutrophils were depleted in both sexes with dexamethasone, this was particularly pronounced in males (1.9 FC higher at t1 and 3.4 FC higher at t2; Extended Data Fig. 9e). Of the two salient neutrophil state alterations, an immature (ARG1^+^ immunosuppressive) state was preferentially expanded with dexamethasone in males (Extended Data Fig. 9e), whereas ISGs were preferentially suppressed (Extended Data Fig. 9f) and IFN^active^ states were depleted in females (Extended Data Fig. 9g,h) at t1 and t2 (Fig. 4h,i). Sexually dimorphic dexamethasone effects on neutrophil maturation kinetics might, in part, explain these alterations. Dynamo-inferred vector fields (predictions of neutrophils’ near-future states) revealed dexamethasone-induced features that were preferentially regulated in females. Dexamethasone was associated with accelerated immature (ARG1^+^ immunosuppressive) neutrophil differentiation at t1 and stunted IFN^active^ neutrophil transitions at t2 (Extended Data Fig. 9i,j). This suggests that the sexually dimorphic effect of dexamethasone might be due to dimorphic alterations of neutrophil maturation, resulting in preferential depletion of IFN^active^ neutrophils concomitant with a lack of immature neutrophil expansion in females.

## Discussion

Surviving SARS-CoV-2 depends on striking a temporal balance between inciting viral clearance immune programs during the early stage and subsequently restraining those same programs at later stages to limit immunity-induced damage. IFN signaling stands at the nexus between anti-viral immunity and overactive effector immune programs that inadvertently compromise tissue function and threaten survival^54^. Our work uncovered a stable neutrophil state with signature downstream IFN signaling that is selectively expanded during late-stage COVID-19 infection. Inborn errors^34^ and suppressed early-stage^6^ IFN signaling predict COVID-19 severity, and increased IFN^active^ neutrophils in females correlated with decreased mortality^55^. Thus, early initiation of IFN therapy has been suggested to mitigate disease severity^56,57^. Given these observations, one might posit that IFN activity in neutrophils represents a concerted host anti-viral program.

Interestingly, immunosuppression with dexamethasone, a corticosteroid known to improve mortality in patients hospitalized with COVID-19 (ref. ^15^), was associated with global alteration of neutrophils as well as suppression of neutrophilic IFN networks and preferential depletion of COVID-19-enriched IFN^active^ neutrophils. These altered neutrophil states shared striking resemblances to bacterial ARDS, suggesting that installation of generalized microbicidal programs ameliorate the overzealous neutrophil responses during COVID-19 (and perhaps during other viral infections). Although neutrophil ISG activation might promote anti-viral immunity during early stages of SARS-CoV-2 infection, sustained IFN activation during late stages (for example, patients admitted to the ICU with severe disease) could drive immunopathology of COVID-19. Indeed, positive correlation between neutrophil type 1 IFN programs and COVID-19 severity^7,58^, paired with our observation that IFN^active^ neutrophils dominate the bronchoalveolar microenvironment during severe COVID-19 (ref. ^11^), support this view.

Another neutrophil state that emerged with COVID-19 (and was absent in healthy controls) was an ARG1^+^ immature and immunosuppressive state with immunomodulatory properties^45–49^. This state was significantly expanded with dexamethasone, suggesting a second route of effect of dexamethasone on both neutrophils and systemic innate immune response. Whether immature neutrophils arise due to enhanced liberation in bone marrow, release of marginated cells or restrained differentiation due to dexamethasone remains to be determined. Although dexamethasone did not appear to increase the frequency of proliferating immature neutrophils, future experiments should interrogate dexamethasone-induced expansion of granulocyte/macrophage progenitors within the bone marrow or hastened liberation of immature neutrophils to explain the expanded pool of immature neutrophils in circulation. Further investigation into direct versus indirect effects of dexamethasone on neutrophils would impart insights into dexamethasone autonomous effects. Immunotherapies supporting innate anti-viral immunity by decoupling IFN-exaggerated neutrophil response while reinforcing suppressor states might limit neutrophil pathogenicity and provide benefit for severe COVID-19.

Our study has three major limitations. First, it is a pragmatic retrospective cohort study and not a randomized controlled trial. During the study enrollment period, dexamethasone became standard of care, leading to a fixed size and sex of the non-dexamethasone group. Non-random allocation and small sample size might inadvertently introduce selection bias and limit generalizability of dexamethasone findings. Second, comparisons against bacterial ARDS, and not another respiratory viral infection, preclude assessment of whether dynamics defined are specific to SARS-CoV-2. Finally, a subset of patients sampled at t1 was discharged from the ICU before t2 collection, precluding estimation of temporal changes.

Several exciting avenues of study remain, including investigating where neutrophil polarization occurs in response to both dexamethasone and COVID-19 infection. Given that pre-neutrophils in marrow become non-mitotic and can enter the bloodstream in an early or immature form (morphologically defined as band cells), we speculate that neutrophil state alterations occur after they enter circulation; however, this needs formal testing. Because neutrophils do not divide, we think it is unlikely that the increase in polarized subsets is due to expansion or replication of pre-existing polarized states that were observed at low numbers in healthy controls. However, there are no definitive data to know if polarized neutrophils arise from distinct lineage-restricted precursor pools in the bone marrow. Defining mechanisms that drive neutrophil state polarization will shed light on whether neutrophil state changes reflect a dynamic continuum or are the result of pre-ordained functional programming and will enable researchers to therapeutically target unwanted neutrophil states or enhance beneficial neutrophil states to combat disease.

## Methods

### Patient enrollment

We recruited six bacterial ARDS (*n* = 5 at t1 and *n* = 4 at t2), eight non-dexamethasone COVID-19 ARDS (*n* = 8 at t1 and *n* = 4 at t2) and six dexamethasone-treated COVID-19 ARDS (*n* = 6 at t1 and *n* = 3 at t2) patients who were admitted to the ICU (Supplementary Table 2). All patients were enrolled after admission to any of the four adult ICUs at South Health Campus, Rockyview General Hospital, Foothills Medical Center or Peter Lougheed Center in Calgary, Alberta, Canada (Extended Data Fig. 1). Patient admission to the ICU was determined by the attending ICU physician based on the need for life-sustaining interventions, monitoring and life support. The research teams did not participate in clinical decisions. Study inclusion required a minimum age of 18 years of age, the ability to provide consent or, for most participants, the ability of a surrogate decision-maker to provide regained capacity consent. All participants required an arterial catheter for blood draws, but the insertion of this catheter was at the discretion of the attending medical team. Patients with COVID-19 ARDS required a positive clinical RNA COVID-19 test before enrollment and evidence of bilateral lung infiltrates and hypoxemia consistent with ARDS. All patients with COVID-19 ARDS were treated with empiric antibiotics. At the time of sample collections, all enrolled patients who were positive for COVID-19 were culture negative for concurrent bacterial infections in the blood, urine and sputum. The bacterial ARDS cohort required a negative COVID-19 test and a definitive microbiological diagnosis of bacterial pneumonia with chest imaging consistent with a diagnosis of ARDS. Patients were excluded from our study if they (1) were on immunosuppressive therapies; (2) had established autoimmune disease; or (3) had active malignancy. Because tocilizumab, remdesivir or any other immunomodulatory agents were not approved for use in patients with severe COVID-19 in Alberta over the time span of this study, participants did not receive these medications. Starting on 1 June 2020, all patients with COVID-19 received dexamethasone (6 mg per day) upon hospital admission, as dexamethasone became the standard of care at that time. Although patients with bacterial ARDS received appropriate antibiotic treatments, none was prescribed immunosuppressive or steroid therapy. All patients with bacterial ARDS had lung infections caused by Gram-positive cocci (four *S. aureus* and two *S. pneumoniae*). To be included, participants were required to have a definitive diagnosis and appropriate consent and samples collected within 72 h of admission to the ICU. t1 refers to the first blood draw, whereas t2 was a repeat blood draw taken 7 d after t1, if the participant remained in the ICU and had an arterial catheter. For each participant, whole blood was collected via the arterial catheter and immediately processed for analysis. Healthy blood donors were recruited by university-wide advertisement and required that participants were (1) not on immunomodulatory medications; (2) were asymptomatic for COVID-19; (3) did not receive vaccination against SARS-CoV-2; and (4) did not have underlying immune disorders.

### Epidemiological analysis

For this study, we used the Alberta provincial eCritical Oracle-based analytics database (Tracer) to query and extract Alberta COVID-19 ICU cases and volumes^59^. Aggregate data from 16 individual adult ICUs were obtained over the study periods. Data for dexamethasone administration could not be captured at an individual level; therefore, we queried the database for patients admitted to the ICU before dexamethasone became standard of care in our province (pre-dexamethasone era: 1 January 2020 to 31 May 2020) versus dexamethasone as standard of care for severe COVID-19 (1 June 2020 to 31 May 2021). Tocilizumab was approved for use in Alberta on 11 March 2021, and a small supply (150 doses) was obtained for patients with severe COVID-19 after this date.

### Human study ethics

All work with humans was approved by the Conjoint Health Research Ethics Board at the University of Calgary (Ethics ID: REB20-0481) and is consistent with the Declaration of Helsinki.

### Serum cytokine assessment

Cytokines, chemokines and soluble cytokine receptors were quantitated on multiplex arrays that included a 65 MILLIPLEX cytokine/chemokine (6Ckine, BCA-1, CTACK, EGF, ENA-78, Eotaxin, Eotaxin-2, Eotaxin-3, FGF-2, Flt-3L, Fractalkine, G-CSF, GM-CSF, GRO, I-309, IFN-α2, IFN-γ, IL-1α, IL-1β, IL-1ra, IL-2, IL-3, IL-4, IL-5, IL-6, IL-7, IL-8, IL-9, IL-10, IL-12 (p40), IL-12 (p70), IL-13, IL-15, IL-16, IL-17A, IL-18, IL-20, IL-21, IL-23, IL-28a, IL-33, IP-10, LIF, MCP-1, MCP-2, MCP-3, MCP-4, MDC, MIP-1α, MIP-1β, MIP-1d, PDGF-AA, PDGF-AB/BB, RANTES, SDF-1α, SDF-1β, sCD40L, SCF, TARC, TGFa, TNFa, TNFb, TPO, TRAIL, TSLP, VEGF) and a 14 MILLIPLEX soluble cytokine (sCD30, sEGFR, sgp130, sIL-1RI, sIL-1RII, sIL-2Ra, sIL-4R, sIL-6R, sRAGE, sTNF RI, sTNF RII, sVEGF R1, sVEGF R2 and sVEGF R3) (Millipore Sigma) on a Luminex 200 luminometer. EDTA plasma samples were collected from each patient by venipuncture after a standard operating protocol and stored at −80 °C until tested. Each run included a full range of calibrators. The Mann–Whitney *U*-test was used to compare groups, and *P* values were adjusted for multiple comparisons using the Holm−Sídak stepdown method with *α* set to 0.05.

### Shotgun proteomics using liquid chromatography with tandem mass spectrometry

The serum of patients with COVID-19 (COVID-19 non-dexamethasone = 9 and COVID-19 dexamethasone = 4) and bacterial ARDS controls (*n* = 6) were collected. The total protein concentrations were determined by Pierce BCA Protein Assay Kit (23225, Thermo Fisher Scientific). A trichloroacetic acid/acetone protocol was used to pellet 100 µg of proteins per sample (14,000*g*, 15 min, 4 °C), followed by air drying for 2 min. Samples were subjected to a quantitative proteomics workflow as per supplier (Thermo Fisher Scientific) recommendations. Samples were reduced in 200 mM tris(2-carboxyethyl)phosphine for 1 h at 55 °C, and reduced cysteines were alkylated by incubation with iodoacetamide solution (50 mM) for 20 min at room temperature. Samples were precipitated by acetone/methanol, and 600 μl of ice-cold acetone was added, followed by incubation at −20 °C overnight. A protein pellet was obtained by centrifugation (8,000*g*, 10 min, 4 °C), followed by acetone drying (2 min). The precipitated pellet was resuspended in 100 μl of 50 mM triethylammonium bicarbonate buffer, followed by tryptase digestion (5 μg of trypsin per 100 μg of protein) overnight at 37 °C. TMT-6plex Isobaric Labeling Reagents (90061, Thermo Fisher Scientific) were resuspended in anhydrous acetonitrile and added to each sample (41 μl of TMT-6plex per 100-μl sample) and incubated at room temperature for 1 h. The TMT labeling reaction was quenched by 2.5% hydroxylamine for 15 min at room temperature. TMT-labeled samples were combined and acidified in 100% trifluoroacetic acid to pH <3.0 and subjected to C18 chromatography (Sep-Pak) according to manufacturer recommendations. Samples were stored at −80 °C before lyophilization, followed by resuspension in 1% formic acid before liquid chromatography with tandem mass spectrometry (LC–MS/MS) analysis.

Tryptic peptides were analyzed on an Orbitrap Fusion Lumos Tribrid mass spectrometer (Thermo Fisher Scientific) operated with Xcalibur (version 4.0.21.10) and coupled to a Thermo Fisher Scientific Easy-nLC (nanoflow liquid chromatography) 1200 system. Tryptic peptides (2 μg) were loaded onto a C18 trap (75 μm × 2 cm; Acclaim PepMap 100, P/N 164946, Thermo Fisher Scientific) at a flow rate of 2 μl min^–1^ of solvent A (0.1% formic acid in LC–MS-grade water). Peptides were eluted using a 120-min gradient from 5% to 40% (5% to 28% in 105 min, followed by an increase to 40% B in 15 min) of solvent B (0.1% formic acid in 80% LC–MS-grade acetonitrile) at a flow rate of 0.3 μl min^–1^ and separated on a C18 analytical column (75 μm × 50 cm; PepMap RSLC C18, P/N ES803A, Thermo Fisher Scientific). Peptides were then electrosprayed using 2.1 kV voltage into the ion transfer tube (300 °C) of the Orbitrap Lumos operating in positive mode. The Orbitrap first performed a full MS scan at a resolution of 120,000 full width at half maximum to detect the precursor ion having a *m*/*z* between 375 and 1,575 and a +2 to +4 charge. The Orbitrap AGC (automatic gain control) and the maximum injection time were set at 4 × 10^5^ and 50 ms, respectively. The Orbitrap was operated using the top speed mode with a 3-s cycle time for precursor selection. The most intense precursor ions presenting a peptidic isotopic profile and having an intensity threshold of at least 2 × 10^4^ were isolated using the quadrupole (isolation window (*m*/*z*) of 0.7) and fragmented using higher-energy C-trap dissociation (38% collision energy) in the ion routing multipole. The fragment ions (MS2) were analyzed in the Orbitrap at a resolution of 15,000. The AGC and the maximum injection time were set at 1 × 10^5^ and 105 ms, respectively. The first mass for the MS2 was set at 100 to acquire the TMT reporter ions. Dynamic exclusion was enabled for 45 s to avoid acquisition of the same precursor ion having a similar *m*/*z* (±10 p.p.m.).

### Leukocyte and lymphocyte isolation

For lymphocyte isolation, whole blood heparinized vacutubes were used. To isolate lymphocytes by immunomagnetic negative selection, 100 μl of Isolation Cocktail and 100 μl of Rapid Spheres (EasySep Direct Human Total Lymphocytes Isolation Kit, 19655, STEMCELL Technologies) were added to 2 ml of whole blood. After mixing and 5-min incubation at room temperature, the sample volumes were topped up to 2.5 ml with 0.04% BSA in PBS. The diluted sample was incubated in the magnet without a lid for 5 min at room temperature, and negatively selected lymphocytes were decanted into a new 5-ml polystyrene tube. Except for the addition of Isolation Cocktail, all steps were repeated once. The final lymphocyte cell suspension was transferred to a 15-ml polypropylene tube, and a volume of 5 ml of 0.04% BSA in PBS was added to the sample. Lymphocytes were precipitated by centrifugation for 5 min at 300*g*; the supernatant was discarded; and cells were resuspended in 5 ml of 0.04% BSA in PBS. This step was repeated, and cells were resuspended in 100 μl of PBS + 0.04% BSA. Cell density was quantified with a hemacytometer; cell viability was assessed with trypan blue staining (T8154, Sigma-Aldrich); and 7,500 live lymphocytes were transferred to a sterile 1.5-ml microcentrifuge tube.

For leukocyte isolation, 1 ml of whole blood from heparin-containing vacutubes was transferred to 5-ml polystyrene round-bottom tubes, and 12 μl of 0.5 M EDTA was added. Next, 2% FBS in PBS (1 ml) and 50 μl of EasySep RBC Depletion spheres (EasySep RBC Depletion Reagent, 18170, STEMCELL Technologies) were added to immunomagnetically deplete red blood cells. After 5 min of magnet incubation at room temperature, cell suspension containing leukocytes was decanted into a new 5-ml polystyrene tube. To ensure complete removal of red blood cells, red blood cell depletion was repeated, and cell suspension containing leukocytes was decanted into a new 15-ml polypropylene tube. Leukocytes were precipitated by centrifugation at 300*g* for 5 min at 20 °C and resuspended in 5 ml of 0.04% BSA in PBS. This step was repeated, and leukocytes were resuspended in 2 ml of 0.04% BSA in PBS. Cell viability and cell density were assessed, and 7,500 live leukocytes were transferred to the microcentrifuge tube containing the lymphocyte cell suspension in a total volume of 50 μl of 0.04% BSA in PBS.

### Immunocytochemistry and immunohistochemistry

Isolated leukocyte and lymphocyte samples were fixed in 4% paraformaldahyde in PBS (0.2 mM and pH 7.4) and spun in a cytocentrifuge (8 min at 300*g*) onto coated slides. Slides were permeabilized and blocked with 10% normal donkey serum in PBS (with 0.5% Triton X-100), and primary antibodies (S100A8/9, Abcam, ab22506; IFITM1, Abcam, ab233545) were incubated at 4 °C overnight, followed by incubation with donkey anti-rabbit Alexa Fluor 488 (Invitrogen, A32790) or anti-mouse Alexa Fluor 555 (Invitrogen, A31570) for 1 h at room temperature. Slides were sequentially stained with CD24 (Abcam, ab202073) on the same slides for 1 h at room temperature, followed by donkey anti-rabbit Alexa Fluor 647 (Invitrogen, A31573). Imaging was done using a VS-120 slide scanner (Olympus), and high-resolution imaging was done using an SP8 spectral confocal microscope (Leica). Image processing was completed in Fiji (version 2.1.0)^60^.

### scRNA-seq library construction, alignment and quality control

A total of 15,000 single cells (containing an equal proportion of leukocytes and lymphocytes) were loaded for partitioning using 10x Genomics NextGEM Gel Bead emulsions (3′ gene expression kit, version 3.1). All samples were processed as per the manufacturer’s protocol (both PCR amplification steps were run 12×). Quality control (QC) of resulting libraries and quantification was performed using TapeStation D1000 ScreenTape assay (Agilent). Sequencing was performed using Illumina NovaSeq S2 and SP 100 cycle dual lane flow cells over multiple rounds to ensure that each sample received at least 32,000 reads per cell. Sequencing reads were aligned using the CellRanger 3.1.0 pipeline^61^ to the standard pre-built GRCh38 reference genome. Samples that passed alignment QC were aggregated into single datasets using CellRanger aggr with between-sample normalization to ensure that each sample received an equal number of mapped reads per cell. Aggregated non-dexamethasone-treated COVID-19 (*n* = 12) and bacterial ARDS (*n* = 9) samples recovered 1,872,659 cells that were sequenced to 38,410 post-normalization reads per cell. Likewise, aggregated COVID-19 samples with (*n* = 9) or without (*n* = 12) dexamethasone recovered 1,748,551 single cells sequenced to 51,415 post-normalization reads per cell. Aggregated healthy samples recovered 19,816 cells, including 1,912 post-QC neutrophils (*n* = 5).

### scRNA-seq computational analyses and workflows

Filtered feature barcode HDF5 matrices from aggregated datasets were imported into the R package Seurat (version 3.9 and version 4) for normalization, scaling, integration, multi-modal reference mapping, Louvain clustering, dimensionality reduction, differential expression analysis and visualization^62^. In brief, cells with abnormal transcriptional complexity (fewer than 500 UMIs, more than 25,000 UMIs or greater than 25% of mitochondrial reads) were considered artifacts and were removed from subsequent analysis. Because granulocytes have relatively low RNA content (due to high levels of RNases), QC thresholds were informed by Xie et. al.^8^. Cell identity was classified by mapping single-cell profiles to the recently published peripheral blood mononuclear cell single-cell joint RNA/CITE-seq multi-omic reference (Azimuth)^63^.

### Annotation of neutrophil states

Because the Azimuth reference does not contain granulocytes that would automate neutrophil annotations within queried datasets, neutrophil clusters were manually annotated by querying known markers (that is, CSF3R, S100A8, S100A9, MMP8, MMP9, ELANE and MPO)^64^ and were corroborated using the R package SingleR^65^. Neutrophil states were defined by grouping unsupervised (Louvain at default resolution) subclusters based on two overlapping criteria: (1) scVelo-inferred neutrophil maturity and (2) by corroborating gene expression and SCENIC-inferred GRN signatures with previous human and rodent neutrophil scRNA-seq studies. Immature neutrophils were defined as CD24^+^ARG1^+^ELANE^+^MPO^+^ATF4^GRN-active^JDP2^GRN-active^ neutrophils^7,8,58,66^ that were reproducibly assigned as ‘root cells’ in scVelo-based latent time pseudo-ordering. IFN^active^ neutrophils were defined by preferential mRNA splicing (positive velocity) and expression of ISGs, such as IFITM1/2, IFIT1/2/3, ISG15/20 and IFI6/27/44/44L^6,55,67^. PG^active^ neutrophils were distinguished by preferential splicing of PTGS2/COX2 (as well as expression for prostaglandin transport LST1)^55^ and included a subset that expressed high levels of IL-1β decoy receptor IL-1R2 (ref. ^42^). Lastly, IL-7R^+^ neutrophils (a small but distinct subset that might be of thymic origin^68^) expressed high levels of ribosomal subunit genes (for example, RPL5/7A/8/13/18/19/23/24/27/P0) that are highly reminiscent of ‘ribosomal^hi^-specific cluster 7’ identified previously^58^.

### Statistical approach for comparing cell proportions

To test whether cell composition was changed due to infection type (COVID-19 versus bacterial ARDS) or treatment group (dexamethasone versus non-dexamethasone), a generalized linear mixed-effects model was employed where infection type and treatment group were considered fixed, and individual patients were considered random effect. Fitting was done with Laplace approximation using the ‘glmer’ function in the ‘lme4’ R package (version 1.1-27.1)^69^, and *P* values were calculated using the R package ‘car’ (version 3.0-11). Box plots comparing cell type composition were generated using the ggplot2 package. Because a subset of patients sampled at t1 was discharged from ICU before t2 collection (non-random or non-ignorable missing data), we limited statistical comparisons to between-group comparisons within one time point (for example, COVID-19 t1 versus bacterial ARDS t1 or dexamethasone-treated t1 versus non-dexamethasone-treated t1) and did not estimate temporal differences across t1 and t2.

### Inferring cell communication networks

Differential cell–cell interaction networks were reconstructed using the Connectome R toolkit version 0.2.2 (ref. ^70^) and CellChat version 1.0.0 (ref. ^71^). In brief, DifferentialConnectome queried Seurat (version 3.9 and version 4) R objects housing datasets integrated by infection type and dexamethasone status to define nodes and edges for downstream network analysis. The total numbers of interactions and interaction strengths were calculated using the compareInteractions function in CellChat. The differential edge list was passed through CircosDiff (a wrapper around the R package ‘circlize’) and netVisual_chord_gene in CellChat to filter receptor ligand edges and generate Circos plots.

### Consensus DEGs and perturbation scores

DEGs were those with an average logFC greater than 0.25 (adjusted *P* < 0.05) as determined by Seurat (version 3.9 and version 4) Wilcoxon rank-sum test. Consensus stacked bars showing cumulative logFCs (colored by individual sample contributions) were generated using the constructConsensus function^7^ for genes exhibiting reproducible changes across patients (>3 for 72-h comparisons and >2 for 7-d comparisons). Gene set enrichment analyses of consensus DEGs were performed using gProfiler’s g:GOSt (*P* value cutoff < 0.05). A cell-state-specific ‘perturbation score’ was calculated to reflect the magnitude of response elicited by factoring in number and cumulative FC of consensus DEGs. Perturbation scores were visualized using Nebulosa (version 1.0.2)-generated density plots^72^.

### Constructing cellular trajectories using RNA velocity

Analysis of neutrophil trajectories was performed by realigning CellRanger count-generated BAMs with the RNA velocity command line tool^27^ using the run10x command and human (GRCh38) annotations. The output loom files containing spliced and unspliced counts were combined to compare neutrophils in COVID-19 with bacterial ARDS controls and dexamethasone-treated with non-treated patients with COVID-19. For both analyses, combined looms were imported into Seurat (version 3.9 and version 4) using the ReadVelocity function in SeuratWrappers version 0.2.0, normalized using SCTransform (version 0.3.2)^73^, reduced and projected onto a UMAP and exported as an H5 file using the SaveH5Seurat function. Counts stored in H5 files were imported, filtered and normalized as recommended in the scVelo (version 0.2.1) workflow^26^. RNA velocities were estimated using stochastic and dynamical models. Because both models yielded similar results, a stochastic model was used as default for all subsequent analyses. Calculations stored in AnnData’s metadata were exported as CSV files, and kernel density lines depicting Velocity-inferred latent time distribution were plotted with ggplot2 (version 3.1.1).

### Gene regulatory network and Gene Ontology enrichment

SCENIC^35^ was employed to infer regulatory interactions between TFs and their targetome by calculating and pruning co-expression modules. In brief, neutrophils were subsetted from scVelo-realigned Seurat (version 3.9 and version 4) object and processed using default and recommended parameters specified in SCENIC’s vignette (https://github.com/aertslab/SCENIC) using the hg19 RcisTarget reference. Regulon activity scores (in ‘3.4_regulonAUC.Rds’, an output of the SCENIC workflow) were added to scVelo object (using the CreateAssayObject function) to jointly project trajectory and TF activity onto the same UMAP embeddings. Consensus stacked bars showing cumulative logFC of AUCell scores for each TF (colored by individual sample contributions) were generated by modifying the constructConsensus function^7^ for the SCENIC assay. The targetome of TFs predicted as drivers of neutrophil states (stored in ‘2.6_regulons_asGeneSet.Rds’) was profiled using g:Profiler’s functional enrichment analysis, and genes intersecting with the INF pathway were plotted using iRegulon (Cytoscape plugin)^74^. Gene Ontology term enrichment analysis was performed using the Seurat (version 3.9 and version 4) DEenrichRPlot function, a wrapper around the Ma’ayan lab’s Enrichr^75^, where DEGs were calculated using the Wilcoxon rank-sum test, and a maximum of 300 genes were provided as input to Enrichr.

### Comparing scRNA-seq findings with published datasets

To test whether dexamethasone-suppressed neutrophil genes at t1 and t2 (Supplementary Table 5) predicted COVID-19 mortality, we repurposed methods described in ref. ^42^ and employed whole blood bulk RNA-seq datasets generated in ref. ^43^ as a validation cohort of 103 samples (where 17 were fatal). In brief, each of the 103 samples was scored by the aggregated expression of dexamethasone-suppressed neutrophil consensus genes at t1 and t2 using Seurat (version 3.9 and version 4) AddModuleScore(). Dexamethasone-suppressed module scores were used as the predictor variable, and 28-d mortality was used as the response variable to construct a receiver operating characteristic (ROC) curve using pROC’s roc() function. To infer bronchoalveolar neutrophil composition in severe and moderate COVID-19 (ref. ^11^) and across bacterial pneumonia and COVID-19 (ref. ^41^), neutrophils (CSF3R^+^, S100A8^+^ and S100A9^+^) captured in BALF scRNA-seq datasets were projected onto our peripheral blood reference using mutual nearest neighbor anchoring (FindTransferAnchors) and the identity transferring (TransferData and AddMetaData) strategy implemented in Seurat version 4 (ref. ^62^).

### COVID neutrophil atlas

To enable intuitive exploration of single-cell datasets, a web portal (http://biernaskielab.ca/COVID_neutrophil or http://biernaskielab.com/COVID_neutrophil) was built using RShiny (version 1.1.0), shinyLP (version 1.1.2) and shinythemes (version 1.1.2) packages.

### Reporting Summary

Further information on research design is available in the Nature Research Reporting Summary linked to this article.

## Online content

Any methods, additional references, Nature Research reporting summaries, source data, extended data, supplementary information, acknowledgements, peer review information; details of author contributions and competing interests; and statements of data and code availability are available at 10.1038/s41591-021-01576-3.

## Supplementary information

Reporting Summary

Supplementary Table 1 Assessment of COVID-19 viral proteins detected in plasma from COVID-19^+^ donors using liquid chromatography and mass spectrometry. ND, not detected. This table shows raw data for plots shown in Fig. 2a.

Supplementary Table 2 Sample size and donor IDs of patients across the three clinical cohorts (bacterial ARDS, non-dexamethasone COVID-19 ARDS and dexamethasone-treated COVID-19 ARDS) at t1 and t2 (tab 1). Clinical and demographic characteristics of all analyzed donors in COVID-19 versus bacterial ARDS (tab 2) and dexamethasone versus non-dexamethasone COVID-19 controls (tab 3) at t1 and t2 after ICU admission. Healthy control demographics are listed in tab 4.

Supplementary Table 3 Targeted list of shotgun proteomics comparing (1) COVID-19 and bacterial ARDS serum or (2) COVID-19 and dexamethasone-treated patients. Tab 1 lists proteins that are significantly different (adjusted *P* < 0.05), and tab 2 lists the proteins that fall outside of the interquartile range (IQR) (the IQR is listed for each comparison). *P* values were determined using a linear mixed-effects model with empirical Bayes moderation as implemented in the MSstatsTMT package. Multiple comparisons were corrected using the Benjamini–Hochberg approach.

Supplementary Table 4 Consensus DEGs in each major cell type in patients with COVID-19 relative to bacterial ARDS 72 h and 7 d after ICU admission. Reported are average logFC of DEGs comparing patients with COVID-19 at t1 (columns C1 and C3–C9) and t2 (columns A3, A5, A6 and A8) to bacterial ARDS controls.

Supplementary Table 5 Consensus DEGs in each major cell type in patients with COVID-19 treated with dexamethasone relative to non-dexamethasone COVID-19 controls t1 and t2 after ICU admission. Reported are average logFC of DEGs comparing patients with dexamethasone at t1 (columns C10–C15) and t2 (columns A10, A12 and A14) to non-dexamethasone-treated COVID-19 controls.

Supplementary Table 6 Sexually dimorphic neutrophil DEGs in patients with COVID-19 treated with dexamethasone relative to non-dexamethasone COVID-19 controls at t1 after ICU admission. Reported are neutrophil transcriptome modulated by dexamethasone in both sexes (columns B–G), in males alone (columns I–N) and in females alone (columns P–S). *P* values were calculated using two-sided *t*-tests adjusted with Bonferroni correction for multiple comparisons.

Supplementary Table 7 SCENIC-predicted targetomes and their Gene Ontology analysis for TFs displayed in Fig. 2.

Supplementary Table 8 Statistical analysis values for Extended Data Figs. 3f, 5g and 9f.

Supplementary Video 1 Dynamo-reconstructed neutrophil vector field topology (left) and animation depicting fate commitments toward COVID-19 and bacterial ARDS-enriched states (right). The color of each digit and the shape of each node shown in the reference image (left) symbolize distinct features of the vector field topology. Half circles represent saddle points; full circles represent stable fixed points; black digits represent absorbing fixed points; red digits represent emitting fixed points; and blue digits represent unstable fixed points.

## Data Availability

eCritical is a secure patient database that is not publicly accessible. Requests for access to patient-related data—de-identified, summary or patient-level data—must be approved by eCritical with an appropriate ethics protocol and might require approval by Alberta Health Services. scRNA-seq datasets are available at the National Center of Biotechnology Information’s Gene Expression Omniobus (which automatically makes Sequence Read Archive deposit) under the following accession number: GSE157789. Single-cell datasets can be further explored on our companion portal at http://biernaskielab.ca/COVID_neutrophil or http://biernaskielab.com/COVID_neutrophil. Velocyto-generated loom files and processed R objects are available for reanalysis from 10.6084/m9.figshare.14330795. Whole blood bulk RNA-seq datasets employed as an independent validation cohort were downloaded from GSE157103. BALF scRNA-seq datasets from severe and moderate COVID-19 were downloaded from GSE145926. Processed BALF scRNA-seq objects from patients with bacterial pneumonia and COVID-19 (archived at GSE167118) were downloaded from the authors’ archive: https://figshare.com/articles/dataset/_/13608734. Mass spectrometry datasets are available at the ProteomeXchange Consortium in the PRIDE partner repository with identifier PXD028429.

## Code availability

All analyses were performed using publicly available software as described in the Methods section. Raw scripts are available upon reasonable request.
