# Supplementary material for: Dexamethasone modulates immature neutrophils and interferon programming in severe COVID-19
Source: Nat Med. 2021 Nov 15;28(1):201–11. doi: 10.1038/s41591-021-01576-3 (PMC8799469; doi:10.1038/s41591-021-01576-3)
Supplement: Supplementary file 1 — Reporting Summary [file 41591_2021_1576_MOESM1_ESM.pdf]

nature research

Corresponding author(s): Dr. Jeff Biernaskie, Dr. Bryan Yipp, Dr. Nicole L. Rosin

Last updated by author(s): Oct 6, 2021

Reporting Summary

Nature Research wishes to improve the reproducibility of the work that we publish. This form provides structure for consistency and transparency in reporting. For further information on Nature Research policies, see our Editorial Policies and the Editorial Policy Checklist.

Statistics

For all statistical analyses, confirm that the following items are present in the figure legend, table legend, main text, or Methods section.

n/a	Confirmed	
☐	☒	The exact sample size (*n*) for each experimental group/condition, given as a discrete number and unit of measurement
☐	☒	A statement on whether measurements were taken from distinct samples or whether the same sample was measured repeatedly
☐	☒	The statistical test(s) used AND whether they are one- or two-sided<br>*Only common tests should be described solely by name; describe more complex techniques in the Methods section.*
☐	☒	A description of all covariates tested
☐	☒	A description of any assumptions or corrections, such as tests of normality and adjustment for multiple comparisons
☐	☒	A full description of the statistical parameters including central tendency (e.g. means) or other basic estimates (e.g. regression coefficient) AND variation (e.g. standard deviation) or associated estimates of uncertainty (e.g. confidence intervals)
☐	☒	For null hypothesis testing, the test statistic (e.g. F, t, r) with confidence intervals, effect sizes, degrees of freedom and P value noted<br>*Give P values as exact values whenever suitable.*
☒	☐	For Bayesian analysis, information on the choice of priors and Markov chain Monte Carlo settings
☒	☐	For hierarchical and complex designs, identification of the appropriate level for tests and full reporting of outcomes
☒	☐	Estimates of effect sizes (e.g. Cohen's *d*, Pearson's *r*), indicating how they were calculated

Our web collection on statistics for biologists contains articles on many of the points above.

Software and code

Policy information about availability of computer code

Data collection

Single-cell RNA-Seq library construction, alignment, and quality control: 10X Genomics NextGEM Gel Bead emulsions (Version 3.1). Illumina NovaSeq S2 and SP 100 cycle dual lane flow cells over multiple rounds to ensure each sample received approximately 32,000 reads per cell. MIlliPLEX cytokine/chemokine and a 14 MilliPLEX soluble cytokine arrays (Millipore Sigma, Oakville, ON, Canada) on a Luminex Model 200 Luminometer (Luminex Corporation, Austin, TX).

Shotgun proteomics was performed using Liquid Chromatography and Mass Spectrometry (LC-MS/MS) using FAIMS Pro (Thermo Fisher Scientific). Briefly, precipitated protein pellet was resuspended in 100 microL of 50mM triethylammonium bicarbonate (TEAB) buffer followed by tryptase digestion overnight at 37°C. TMT-6plex™ Isobaric Labeling Reagents (90061, Thermo Fisher) were resuspended in anhydrous acetonitrile and added to each sampleand incubated at room temperature for 1h. The TMT labeling reaction was quenched by 2.5% hydroxylamine for 15min at room temperature. TMT labeled samples were combined and acidified in 100% trifluoroacetic acid to pH < 3.0 and subjected to C18 chromatography (Sep-Pak) according to manufacturer recommendations. Samples were stored at -80°C before lyophilization, followed by resuspension in 1% formic acid before liquid chromatography and tandem mass spectrometry analysis. Tryptic peptides were analyzed on an Orbitrap Fusion Lumos Tribrid mass spectrometer (Thermo Scientific) operated with Xcalibur (version 4.0.21.10) and coupled to a Thermo Scientific Easy-nLC (nanoflow liquid chromatography) 1200 System. Tryptic peptides were loaded onto a C18 trap (75microm x 2cm; Acclaim PepMap 100, P/N 164946; ThermoFisher). The fragment ions (MS2) were analyzed in the Orbitrap at a resolution of 15,000. The AGC and the maximum injection time were set at 1 x 105 and 105ms, respectively. The first mass for the MS2 was set at 100 to acquire the TMT reporter ions. Dynamic exclusion was enabled for 45 seconds to avoid of the acquisition of same precursor ion having a similar m/z (plus or minus 10ppm).

Imaging was done using a VS-120 slide scanner (Olympus) and high resolution image imaging was done using an SP8 spectral confocal microscope (Leica). Image processing was completed in Fiji (V.2.1.0).

Full data collection is described in the Methods section.

Data analysis

Single-cell RNA-Seq library construction, alignment, and quality control: Sequencing reads were aligned using CellRanger 3.1.0 pipeline to the standard pre-built GRCh38 reference genome.

Single-cell RNA-Seq computational analyses and workflows: R package Seurat (v.3.9, v.4) for normalization, scaling, integration, multi-modal reference mapping, louvain clustering, dimensionality reduction, differential expression analysis, and visualization. Fitting was done with Laplace approximation using the 'glmer' function in the 'lme4' R package (v.1.1-27.1) and p-values were calculated using the R package 'car' (v.3.0-11). Differential cell-cell interaction networks were reconstructed using CellChat v1.0.0.

Consensus DEGs and perturbation scores: Differentially expressed genes (DEGs) were those with an average log fold change (FC) greater than 0.25 (p-adjusted < 0.05) as determined by Seurat's Wilcoxon rank-sum test. Consensus stacked bars were generated using constructConsensus function for genes exhibiting reproducible changes across patients (>3 for 72-hour comparisons, > 2 for 7-day comparisons). Gene Set Enrichment analyses were performed using gProfiler's g:GOSt (p-value cutoff <0.05). Perturbation scores were visualized using Nebulosa v.1.0.2.

Constructing cellular trajectories using RNA velocity: RNA velocity command-line tool using the run10x command and human (GRCh38) annotations. Files read into Seurat (v.3.9, v.4) using the ReadVelocity function in SeuratWrappers v.0.2.0, normalized using SCTransform v.0.3.2, reduced and projected onto a UMAP, and exported as a .h5 file using the SaveH5Seurat function. RNA velocities were estimated using stochastic and dynamical models. Since both models yielded comparable results, stochastic model was used as default for all subsequent analyses.

Gene Regulatory Network reconstruction: Single-cell regulatory network inference and clustering (SCENIC) was employed to infer regulatory interactions between transcription factors (TFs) and their targetome by calculating and pruning co-expression modules. SCENIC repository is available at: https://github.com/aertslab/SCENIC. Targetome of TFs predicted as drivers of neutrophil states (stored in '2.6_regulons_asGeneSet.Rds') was profiled using g:Profiler's functional enrichment analysis and genes intersecting with the Interferon pathway were plotted using iRegulon (Cytoscape plugin).

Epidemiological analysis: We used the Alberta provincial eCRITICAL oracle-based analytics database (Tracer) to query and extract Alberta COVID-19 ICU cases and volumes for this study. Aggregate data from sixteen individual adult ICUs was obtained over the study periods. The administration of dexamethasone was not possible to capture at an aggregate level; therefore, we queried the database for patients admitted to ICU prior to dexamethasone becoming standard of care in our Province (pre-dexamethasone era; January 2020 till May 31st, 2020) versus dexamethasone as standard of care for severe COVID-19 (June 1st, 2020, till May 31st, 2021).

Shotgun proteomics using Liquid Chromatography and Mass Spectrometry (LC-MS/MS): Samples were subjected to a quantitative proteomics workflow as per supplier (Thermo Fisher) recommendations using tryptase digestion (5microgram trypsin per 100microgram of protein).

COVID Neutrophil Atlas: RShiny v1.1.0, shinyLP v.1.1.2, and shinythemes v.1.1.2 packages.

Full data analysis is described in the Methods section.

For manuscripts utilizing custom algorithms or software that are central to the research but not yet described in published literature, software must be made available to editors and reviewers. We strongly encourage code deposition in a community repository (e.g. GitHub). See the Nature Research guidelines for submitting code & software for further information.

Data

Policy information about availability of data

All manuscripts must include a data availability statement. This statement should provide the following information, where applicable:

- Accession codes, unique identifiers, or web links for publicly available datasets
- A list of figures that have associated raw data
- A description of any restrictions on data availability

eCRITICAL is a secure patient database that is not publicly accessible. Requests for access to patient related data, either de-identified, summary or patient level data must be approved by eCRITICAL with an appropriate ethics protocol and may require approval by Alberta Health Services. Single cell RNA-Seq datasets are available at NCBI GEO (which automatically makes SRA deposit) at the following accession: GSE157789. Single-cell datasets can be further explored on our companion portal at http://biernaskielab.ca/COVID_neutrophil or http://biernaskielab.com/COVID_neutrophil. Velocyto-generated LOOM files and processed R objects are available for reanalysis from: http://doi.org/10.6084/m9.figshare.14330795. Whole blood bulk RNA-Seq datasets employed as an independent validation cohort were downloaded from GSE157103. BALF scRNA-Seq datasets from severe and moderate COVID-19 were downloaded from GSE145926. Processed BALF scRNA-Seq objects from patients with bacterial pneumonia and COVID-19 (archived at GSE167118) were downloaded from authors' archive: https://figshare.com/articles/dataset/_/13608734. Mass spectrometry datasets are available via ProteomeXchange Consortium via the PRIDE partner repository with identifier PXD028429.

Field-specific reporting

Please select the one below that is the best fit for your research. If you are not sure, read the appropriate sections before making your selection.

☒ Life sciences ☐ Behavioural & social sciences ☐ Ecological, evolutionary & environmental sciences

For a reference copy of the document with all sections, see nature.com/documents/nr-reporting-summary-flat.pdf

Life sciences study design

All studies must disclose on these points even when the disclosure is negative.

Sample size	Sample size for the non-Dexamethasone-treated patients was restricted due to Dexamethasone becoming standard of care worldwide for ICU patients. Number of dexamethasone-treated samples was pragmatically determined to roughly match their non-Dexamethasone counterpart. The unbiased nature of our interrogation precluded a priori sample size estimation as expected effect size could not be approximated.<br><br>Aggregated non-dexamethasone-treated COVID-19 (n = 12 samples) and bacterial ARDS (n = 9 samples) recovered 1,872,659 cells that were sequenced to 38,410 post-normalization reads per cell. Likewise, aggregated COVID-19 samples with (n = 9 samples) or without (n = 12 samples) dexamethasone recovered 1,748,551 single cells sequenced to 51,415 post-normalization reads per cell. Aggregated healthy samples recovered 19,816 cells, including 1,912 post-QC neutrophils (n = 5 donors).
Data exclusions	We excluded 1 sample (patient ID: C2) from scRNA-Seq analyses as this was collected from a donor who suffered a stroke and tested positive for COVID-19. The patient did not have respiratory issues and spent only 1 night in ICU before being transfered to the stroke ward. The decision to exclude was based strictly on a-priori clinical characteristics. To be comprehensive, we have uploaded this sample to GEO (GSM4775024) and have made a note that this donor was excluded from downstream analyses.
Replication	Sequencing of patient samples happened between April 1st 2020 - Oct 30th 2020. As a result, multiple technical batches were generated by different individuals. Our analyses are based on merging these datasets using an MNN-based batch correction to avoid potential technical biases. Immunohistochemistry experiments were successfully replicated across two separate batches.<br><br>Differential gene expression analyses are presented as consensus stacked bars showing cumulative log fold changes (colored by individual sample contributions) that were generated using constructConsensus function for genes exhibiting reproducible changes across patients (>3 for 72-hour comparisons, > 2 for 7-day comparisons).
Randomization	Allocating patients into experimental groups (i.e. COVID-19 vs Bacterial ARDS) was not feasible.
Blinding	Patient samples were obtained during the first wave of COVID-19 patients in Calgary, Alberta, Canada. All analyses were performed by personnel who were blinded to the patient status.

Reporting for specific materials, systems and methods

We require information from authors about some types of materials, experimental systems and methods used in many studies. Here, indicate whether each material, system or method listed is relevant to your study. If you are not sure if a list item applies to your research, read the appropriate section before selecting a response.

Materials & experimental systems

n/a	Involved in the study	
☐	☒	Antibodies
☒	☐	Eukaryotic cell lines
☒	☐	Palaeontology and archaeology
☒	☐	Animals and other organisms
☐	☒	Human research participants
☒	☐	Clinical data
☒	☐	Dual use research of concern

Methods

n/a	Involved in the study	
☒	☐	ChIP-seq
☒	☐	Flow cytometry
☒	☐	MRI-based neuroimaging

Antibodies

Antibodies used	Isolated leukocyte and lymphocyte samples were fixed in 4% paraformaldahyde in PBS (0.2mM and pH7.4), and spun in a cytocentrifuge (8min at 300g) onto coated slides. Pathological lung sections (FFPE fixed and sectioned at 5um) were deparaffinized in Slide Brite (Fisher Scientific NC968653) and rehydrated. Slides were permeabilized and blocked with 10% normal donkey serum in PBS (with 0.5% triton X-100), primary antibodies (full list presented below) were incubated at 4oC overnight, followed by incubation with secondary antibodies (full list presented below) for 1h at room temperature (RT). Cytospun slides were sequentially stained with CD24 (Abcam ab202073) on the same slides for 1h at RT, followed by donkey anti-rabbit-Alexa647 (Invitrogen A31573).<br><br>Primary antibodies:<br>S100A8/9 (Abcam ab22506), 1:250<br>IFITM1 (Abcam ab233545), 1:250<br>CD24 (Abcam ab202073), 1:250<br><br>Secondary antibodies:<br>donkey anti-rabbit-Alexa488 (Invitrogen A32790), 1:500<br>donkey anti-mouse-Alexa555 (Invitrogen A31570), 1:500<br>donkey anti-rabbit-Alexa647 (Invitrogen A31573), 1:500
Validation	All primary antibodies were validated by their respective manufacturers and individually tested and titrated on relevant positive and

Validation

negative biological controls by the investigators. Representative immunofluorescence images showing positive and negative controls is shown in Extended Data Figure 10.

IFITM1 (Abcam ab233545) validated by confocal imaging.
CD24 (Abcam ab202073) validated by confocal imaging.
S100A8/9 (Abcam ab22506) validated by confocal imaging.

Human research participants

Policy information about studies involving human research participants

Population characteristics

Study inclusion required a minimal age of 18, the ability to provide consent, or for most participants, the ability of a surrogate decision maker to provide regained capacity consent. All participants required an arterial catheter for blood draws, but the insertion of this catheter was at the discretion of the attending medical team. Participants required a positive clinical RNA COVID-19 test prior to enrollment, and evidence of bilateral lung infiltrates and hypoxemia consistent with ARDS. At the time of sample collections, all COVID-19+ enrolled individuals were culture negative for concurrent bacterial infections in the blood, urine, and sputum. The bacterial ARDS cohort required a negative COVID-19 test and a definitive microbiological diagnosis of bacterial pneumonia with chest imaging consistent with a diagnosis of ARDS. Patients were excluded from our study if they: 1. were on immunosuppressive therapies, 2. had established autoimmune disease, or 3. had active malignancy. Since tocilizumab or other immunomodulatory agents were not approved for use in patients with severe COVID-19 in Alberta over the timespan of this study, none of them received these medications. While bacterial sepsis patients received appropriate antibiotic treatments, none were prescribed immunosuppressive or steroid therapy. All bacterial sepsis patients had lung infections caused by gram-positive cocci (4 Staphylococcus aureus and 2 Streptococcus pneumoniae). Participants were required to have a definitive diagnosis and appropriate consent and samples collected within 72hrs of admission to the ICU in order to be included. Timepoint 1 (T1) refers to the first blood draw, while T2 was a repeat blood draw taken 7 days after T1, if the participant remained in the ICU, and had an arterial catheter. For each participant, whole blood was collected via the arterial catheter and immediately processed for analysis. Healthy blood donors were recruited by university-wide advertisement and required that participants were: 1. not on immunomodulatory medications, 2. were asymptomatic for SARS-CoV-2, 3. did not receive vaccination against SARS-CoV-2, and 4. did not have underlying immune disorders.

Our study recruited 6 Bacterial ARDS (3 Males, 3 Females), 8 non-dexamethasone-treated COVID-19 (5 Males, 3 Females), 6 dexamethasone-treated COVID-19 (4 Males, 2 Females), and 5 Healthy (3 Males, 2 Females) donors. Age, ethnicity and clinical characteristics of enrolled individuals is provided in our Supplementary Table 2.

Recruitment

All patients were enrolled following admission to any of the four adult intensive care units at South Health Campus, Rockyview General Hospital, Foothills Medical Center or Peter Lougheed Center in Calgary, Alberta, Canada. These units serve the entire city representing all communities and diverse groups. Patient admission to the ICU was determined by the attending ICU physician based on the need for life sustaining interventions, monitoring and life-support. The research teams did not participate in clinical decisions, including diagnosis and treatment.

Potential recruitment biases include: patients without a surrogate decision maker could not be consented for enrollment and catchment area was limited to hospitals in Calgary, Alberta. As a result, participants without surrogate or family may be under represented or absent in our analysis, however it is not clear how this factor associates with underlying medical conditions, socioeconomic factors, ethnicity, sex or gender.

Ethics oversight

This study was approved by the Conjoint Health Research Ethics Board (CHREB) at the University of Calgary (Ethics ID: REB20-0481) and is consistent with and the Declaration of Helsinki.

Note that full information on the approval of the study protocol must also be provided in the manuscript.
